# Comparative analysis of various machine learning algorithms to predict strength properties of sustainable green concrete containing waste foundry sand

**DOI:** 10.1038/s41598-024-65255-2

**Published:** 2024-06-25

**Authors:** Muhammad Faisal Javed, Majid Khan, Muhammad Fawad, Hisham Alabduljabbar, Taoufik Najeh, Yaser Gamil

**Affiliations:** 1 Department of Civil Engineering, GIK Institute of Engineering Sciences and Technology, Swabi, 23640 Pakistan; 2 Western Caspian University, Baku, Azerbaijan; 3 Civil Engineering Department, COMSATS University Islamabad, Abbottabad Campus, 22060 Pakistan; 4 Silesian University of Technology, Gliwice, Poland; 5 Budapest University of Technology and Economics Hungary, Budapest, Hungary; 6 Department of Civil Engineering, College of Engineering in Al-Kharj, Prince Sattam bin Abdulaziz University, Al-Kharj, 11942 Saudi Arabia; 7 Operation and Maintenance, Operation, Maintenance and Acoustics, Department of Civil, Environmental and Natural Resources Engineering, Luleå University of Technology, Lulea, Sweden; 8 Department of Civil Engineering, School of Engineering, Monash University Malaysia, Jalan Lagoon Selatan, Bandar Sunway, Selangor, 47500 Malaysia

**Keywords:** Waste foundry sand, Strength characteristics, Machine learning, SHAP analysis, Waste management, Engineering, Mathematics and computing

## Abstract

The use of waste foundry sand (WFS) in concrete production has gained attention as an eco-friendly approach to waste reduction and enhancing cementitious materials. However, testing the impact of WFS in concrete through experiments is costly and time-consuming. Therefore, this study employs machine learning (ML) models, including support vector regression (SVR), decision tree (DT), and AdaBoost regressor (AR) ensemble model to predict concrete properties accurately. Moreover, SVR was employed in conjunction with three robust optimization algorithms: the firefly algorithm (FFA), particle swarm optimization (PSO), and grey wolf optimization (GWO), to construct hybrid models. Using 397 experimental data points for compressive strength (CS), 146 for elastic modulus (E), and 242 for split tensile strength (STS), the models were evaluated with statistical metrics and interpreted using the SHapley Additive exPlanation (SHAP) technique. The SVR-GWO hybrid model demonstrated exceptional accuracy in predicting waste foundry sand concrete (WFSC) strength characteristics. The SVR-GWO hybrid model exhibited correlation coefficient values (R) of 0.999 for CS and E, and 0.998 for STS. Age was found to be a significant factor influencing WFSC properties. The ensemble model (AR) also exhibited comparable prediction accuracy to the SVR-GWO model. In addition, SHAP analysis revealed an optimal content of input variables in the concrete mix. Overall, the hybrid and ensemble models showed exceptional prediction accuracy compared to individual models. The application of these sophisticated soft computing prediction techniques holds the potential to stimulate the widespread adoption of WFS in sustainable concrete production, thereby fostering waste reduction and bolstering the adoption of environmentally conscious construction practices.

## Introduction

The construction sector stands at a pivotal juncture where embracing sustainability is not just an option but a necessity. Extensive research has investigated the compelling reasons this sector should forge ahead in its commitment to sustainability. Notably, the construction industry significantly contributes to energy consumption, waste generation, resource depletion, and greenhouse gas emissions. Addressing these environmental concerns is paramount, and the construction sector holds immense potential to minimize its ecological footprint through sustainable practices. A key focus is on resource efficiency, as this industry heavily relies on natural resources like water, raw materials, and energy. By embracing sustainable approaches such as material recycling and optimized energy usage, the industry can significantly reduce resource consumption and waste generation^1,2^. To address sustainability concerns in the construction industry, environment-friendly materials are produced by incorporating various types of waste or recycled materials in cementitious composites, either fully or partially substituting the main elements of concrete. For instance, supplementary cementitious materials (SCMs) such as silica fume^3^, fly ash^4,5^, rice husk ash^6^, and blast furnace slag^7,8^ are pozzolanic materials that contain rich silica content. Other sustainable concretes are those incorporating recycled aggregate^9–11^, glass sands^9^, waste foundry sand (WFS)^12,13^, tire rubber^14,15^, and ceramic^16^.

WFS, a by-product of metal foundries, is increasingly being used as a substitution for fine aggregate in the production of environmentally friendly concrete. Metal foundries generate large quantities of waste materials, with approximately 70% of the weight comprising WFS^17^. The escalating cost of landfilling WFS, ranging from approximately US$135 to $675 per ton, renders it economically impractical for the industries. Furthermore, WFS poses environmental hazards because it contains phenols, zinc, lead, cadmium, and iron remnants^17,18^. The current practices of disposing of WFS in landfills pose significant economic and environmental threats^19^. Experimental studies have demonstrated that waste foundry sand concrete (WFSC) maintains comparable mechanical properties to the control concrete when the fine aggregate is substituted with WFS in the 15–20% range, but a declining pattern is observed with more additions. Other studies have stated a decrease in strength properties beyond a 10% substitution level^20^. This behavior is influenced by multiple variables such as WFS composition, mix proportions, percentage, and concrete ingredients' physical characteristics^21,22^. Singh and Siddique^23^ observed that beyond a 15% inclusion of WFS, there was no substantial enhancement in strength, likely due to the increase in surface area of fine particles. This phenomenon potentially resulted in diminished water-cement gel within the matrix, consequently leading to insufficient binding^23–25^. The decline in strength can be attributed to the matrix's inadequate workability and the presence of binders, namely, the fine carbon and clay powder in the WFS^26,27^. These binders adhere to sand particles, impeding the formation of a robust bond between the cement paste and the aggregate. Siddique and Kadri^28^ observed that incorporating a mineral admixture, such as metakaolin into WFS-containing concrete resulted in strength improvement. Furthermore, Kaur et al.^29^ observed that the introduction of fungal-treated WFS led to strength enhancement, attributed to the filling of concrete pores by fungal spores or biominerals deposited within the cement-sand matrix. Moreover, it is widely recognized that a low water-cement ratio contributes to greater concrete strength. However, incorporating WFS into concrete offers minimal benefits when the water-cement ratio is below 0.50^30^. Salokhe et al.^30^ determined that concrete incorporating WFS sourced from ferrous foundries exhibited superior performance compared to concrete containing non-ferrous WFS in terms of strength enhancement. Incorporating both types of sand resulted in compact concretes with a 20% replacement. While considerable literature exists on WFSC, conducting experimental testing to optimize WFSC can be both time-consuming and costly. Therefore, the development of prediction models that correlate influential parameters and the strength properties of WFSC can effectively address the challenges associated with expensive testing procedures. Moreover, such models can facilitate the sustainable reuse of WFS in the industry. To achieve this, utilizing machine learning (ML) techniques proves to be highly beneficial and relevant.

**Figure 1:**
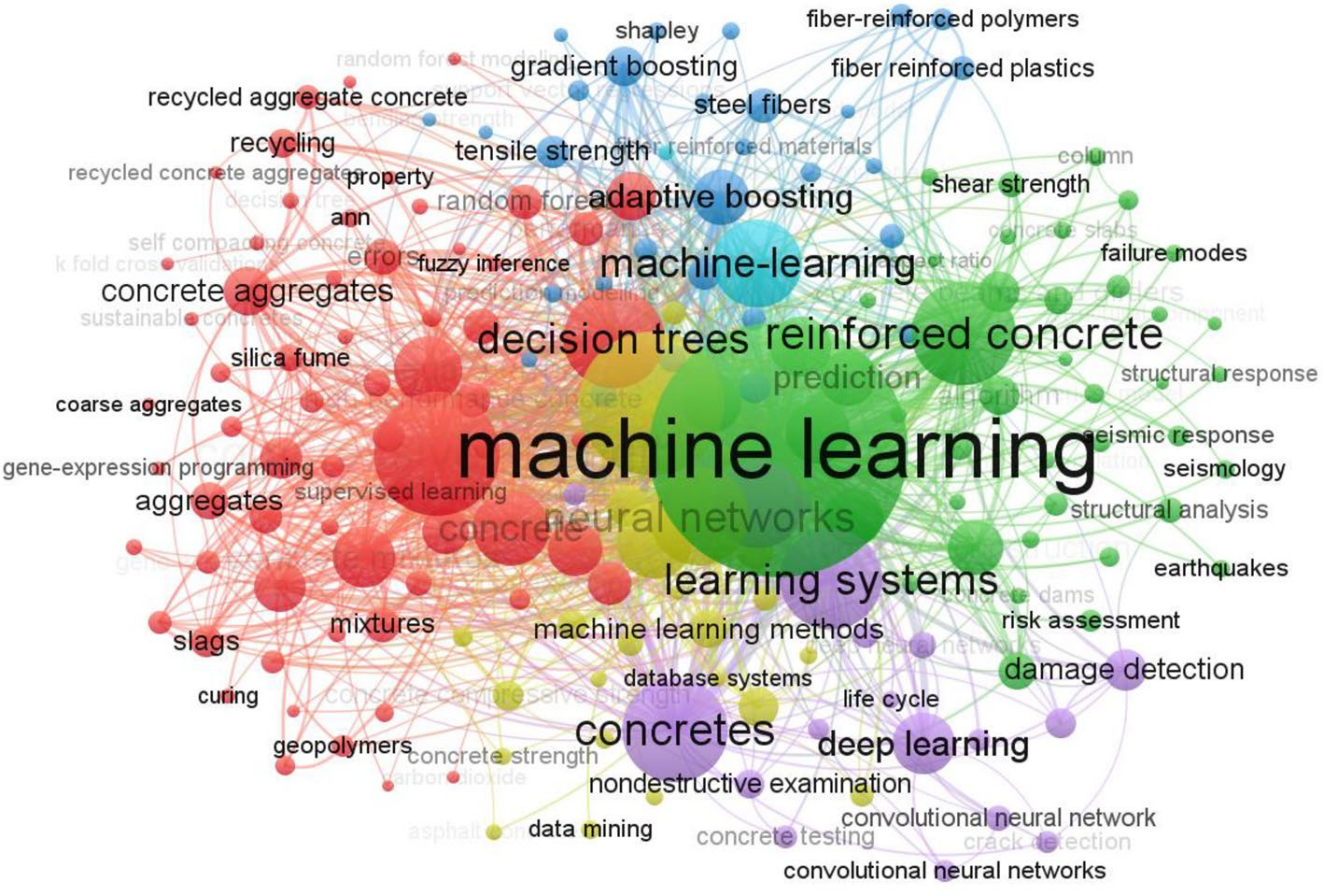
Scientometric analysis of ML applications in construction materials.

Due to the advancement of AI, various soft-computing approaches have been utilized to forecast the characteristics of various types of concrete. For instance, ML methods have been used for predicting properties of recycled aggregate concrete^31,32^, fiber-reinforced concrete^33^, carbon fiber-reinforced concrete^34,35^, geopolymer concrete^36,37^, and concrete containing SCMs such as slag, fly ash, and silica fume^38–40^, as shown in Fig. 1. Among ML techniques, artificial neural networks (ANN)^41–43^, support vector regression (SVR)^44^, genetic engineering programming (GEPs)^13,45,46^, and decision trees (DT)^47,48^ have been commonly utilized. Based on the literature review, multiple studies used ML methods for the estimation of characteristics of WFSC. Iqbal et al.^12^ used the GEP method to forecast the elastic modulus and split tensile strength of WFSC. The GEP approach achieved higher accuracy in estimating the target properties of WFSC. Moreover, Chen et al.^13^ employed both GEP and MEP to forecast the properties of WFSC and reported higher accuracy of the prediction models. The MEP and GEP methods had limitations in their ability to incorporate certain divergent datasets during model development, thus limiting their applicability range. However, to optimize the performance of the models, it is imperative to eliminate the datasets that exhibited significant deviations. Furthermore, genetic algorithms encode a single expression in their programs and are more suitable for relatively simple relationships between input and output^49^. Furthermore, Behnood and Golafshani^50^ also employed the M5P technique to predict the split tensile strength (STS), compressive strength (CS), elastic modulus (E), and flexural strength (FS) of WFSC. The models proposed by the authors demonstrated high precision and enabled the derivation of reliable estimates. Similarly, Amlashi et al.^51^ utilized the ANN model for forecasting the characteristics of WFSC and reported better accuracy of the ANN method to estimate the output. However, ANN models are often called "black boxes" due to their inherent complexity and opacity. The black-box nature of ANNs refers to the challenge of understanding these models' internal workings and decision-making processes^52^. Unlike traditional algorithms, where the steps and rules are explicitly defined, ANNs learn patterns and relationships from data through interconnected layers of neurons. This complexity makes it difficult to interpret how the network arrives at its predictions or decisions^53^. The network's internal representations and transformations of the input data are not easily understandable or explainable. While ANNs have shown remarkable performance in various applications, their lack of interpretability poses challenges in critical domains where transparency is necessary. Above all, the mentioned studies used to predict WFSC’s characteristics are individual or single learning techniques. In contrast, by leveraging the collective intelligence of multiple models, ensemble methods can often outperform individual methods in terms of accuracy, robustness, and generalization^54–56^. Moreover, hybrid models combine the strengths of individual algorithms with optimization techniques to provide excellent prediction models. However, they may be more computationally intensive and require additional model training and combining steps^57^.

Accordingly, this study considered single, ensemble, and hybrid models to predict the properties of WFSC. Ensemble learning (EL) models are developed by combining multiple algorithms to leverage their diverse potentials. By utilizing a mixing mechanism, EL models can achieve higher accuracy and resilience compared to individual algorithms. ELA takes advantage of the strengths of multiple unique algorithms and combines them to enhance accuracy. The approach integrates multiple single learning methods to improve overall predictive performance^57–63^. One popular ensemble method is bagging (Bootstrap Aggregating). Bagging approach considers training multiple models independently on a training dataset, typically using the same learning algorithm. Each model is trained on random data points of the original data^64^. The ultimate prediction is derived by calculating the average of all individual model predictions. Boosting is another widely used ensemble method that sequentially trains models^58^. In boosting, models are trained iteratively, and each successive model in the boosting framework is designed to leverage the errors made by preceding models^65^. The ultimate prediction is achieved by aggregating the predictions with the assigned weights of all models. Boosting methods, such as gradient boosting and AdaBoost, can effectively handle complex datasets and are particularly adept at handling class imbalance problems^38^. In addition to single and ensemble models, hybrid models were also explored in this study to predict the properties of WFSC. Hybrid models combine the strengths of individual algorithms, such as support vector regression (SVR) or neural networks, with optimization techniques like particle swarm optimization (PSO), firefly algorithm (FFA), and grey wolf optimization (GWO). By integrating diverse methodologies, hybrid models aim to enhance predictive accuracy and robustness, offering a more comprehensive approach to addressing the complexities of WFSC prediction tasks.

Given the discussion so far, it is evident that there is a lack of robust and practical machine learning approaches for modeling the characteristics of WFSC. Hence, the main goals of this study are to address these gaps by (i) collecting an extensive data set available on STS, E, and CS published studies, (ii) developing individual ML models (DT, SVR) and EL model (AdaBoost), and hybrid models (SVR-FFA, SVR-PSO, SVR-GWO) (iii) comparative analysis of individual, ensemble, and hybrid models, and (vi) SHAP interpretation of the developed models to unveiled the reasoning and logic behind the ML models prediction.

## Theory of the selected ML algorithms

### Decision tree (DT)

**Figure 2:**
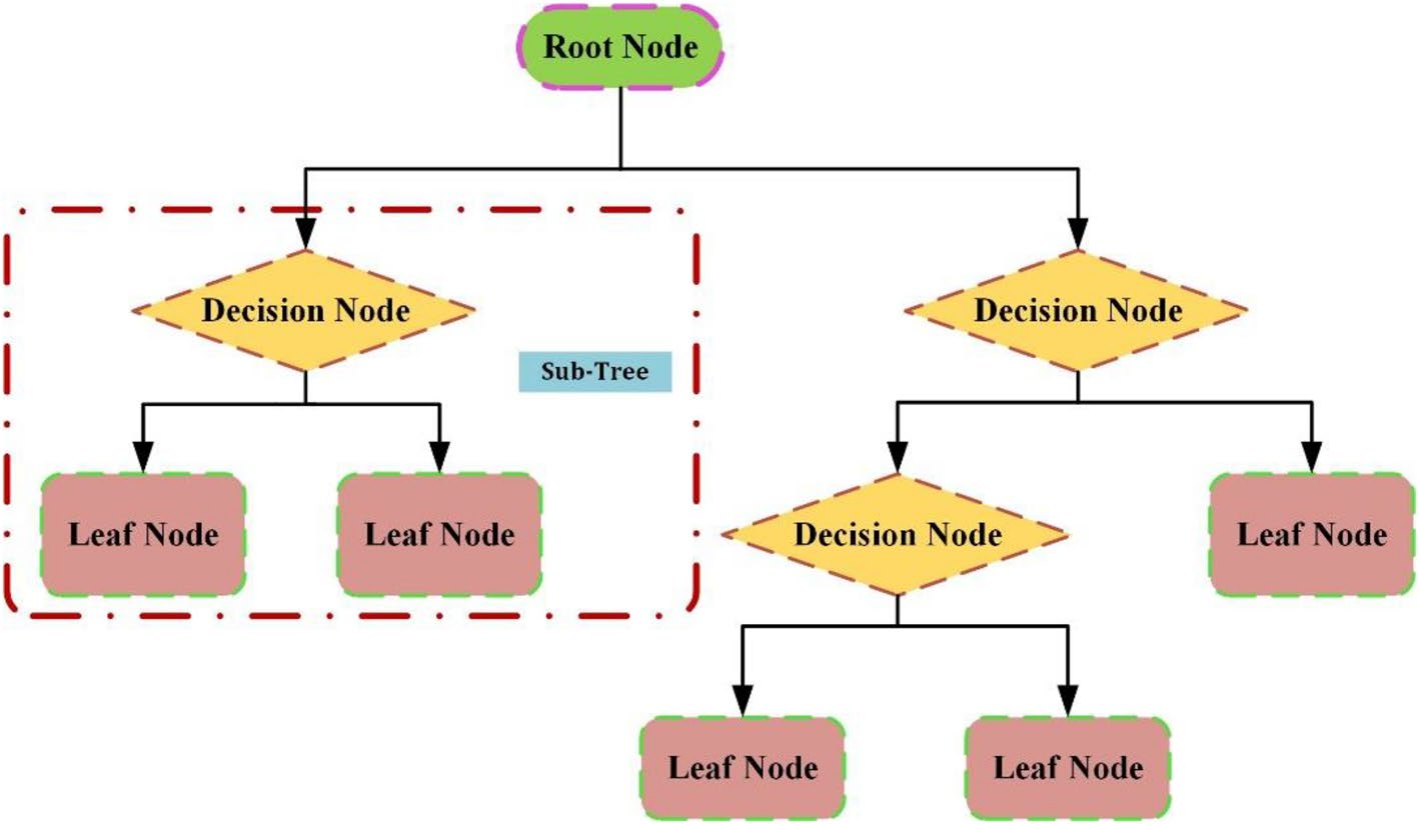
Flowchart of decision tree algorithm.

Due to its flexibility in capturing complex non-linear relationships and ease of interpretation, the decision tree algorithm is widely utilized in various studies^59^. The DT algorithm is a widely employed ML method that builds a predictive model organized in a hierarchical tree shape, representing decisions and their possible consequences^60^. Decision trees are highly effective as they closely mimic the intuitive decision-making process of humans, resulting in enhanced understandability and interpretability. The structure of a decision tree consists of branches and nodes, as shown in Fig. 2. The root node represents the initial decision, and subsequent nodes represent the decisions made at each step. The branches represent the possible outcomes of each decision, leading to different paths or leaves in the tree, representing the final prediction or outcome^61^. The decision tree algorithm aims to create an optimal tree structure by selecting the most informative features to split the data at each node. One of the advantages of the DT model is its interpretability. The tree-like structure allows an understanding of the modeling process and the reasoning behind the prediction. Nonetheless, the DT model is prone to overfitting, particularly when the tree becomes too complicated, or the data contains noise. To mitigate this issue, techniques like pruning or setting a minimum number of instances per leaf can be applied^62^.

### Support vector regression (SVR)

**Figure 3:**
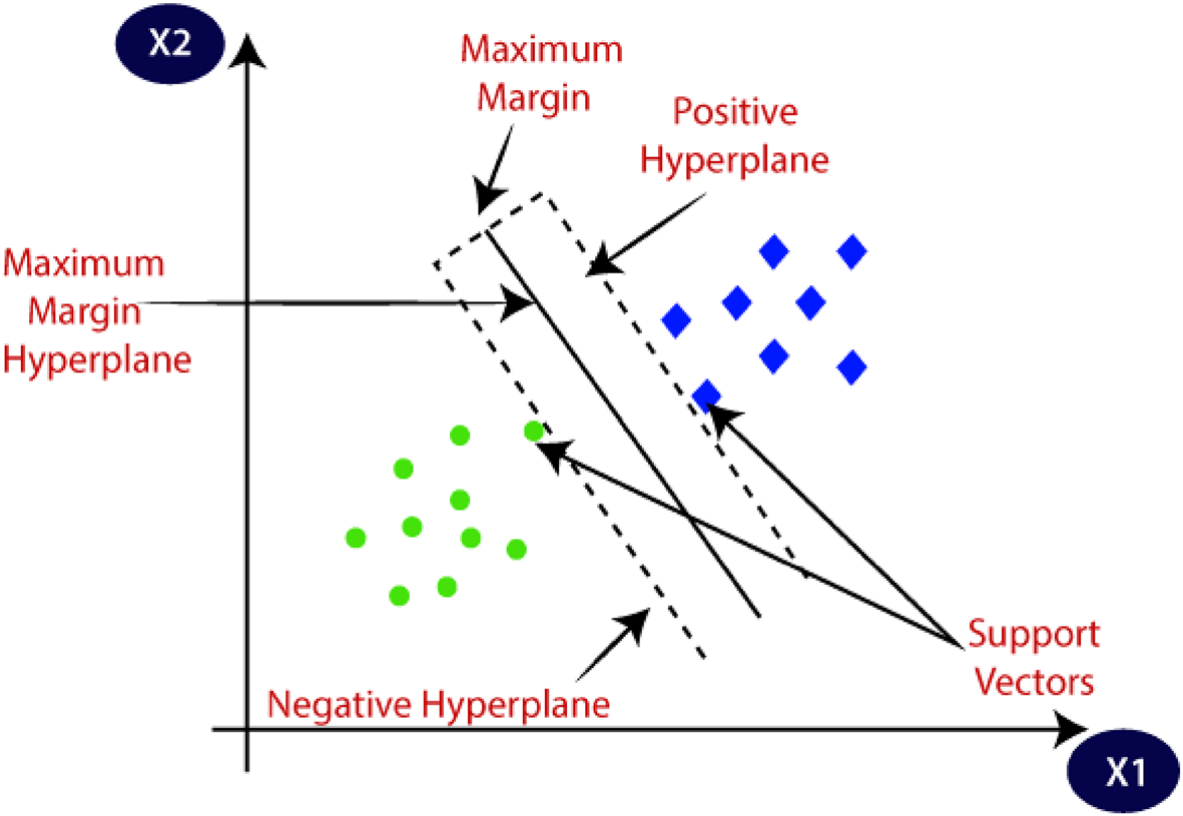
Hyperplane margins for SVR with samples of two classes.

The SVR is a highly effective ML algorithm known for its potential in capturing complex non-linear relationships, making it a favored choice for prediction tasks. Employing feature space transformation, SVR excels in scenarios where data cannot be linearly separated, aiming to identify the optimal hyperplane for maximum separation of data points into distinct classes (Fig. 3). By maximizing the margin, SVR enhances its generalization capability and resilience when encountering unseen data^63^. Utilizing kernel functions like polynomial, linear, radial basis function, and sigmoid, SVR can handle complex decision boundaries, providing flexibility in modeling complex datasets^64,65^. The regularization parameter, denoted as C, plays a critical role, with lower values offering a wider margin but potentially higher misclassification, while higher values of C reduce the margin to improve classification accuracy, albeit with a risk of overfitting. Though computationally expensive and sensitive to parameter and kernel function selection, advancements in optimization algorithms have mitigated these challenges, making SVR a widely adopted effective ML technique^66–68^.

### AdaBoost regressor (AR)

**Figure 4:**
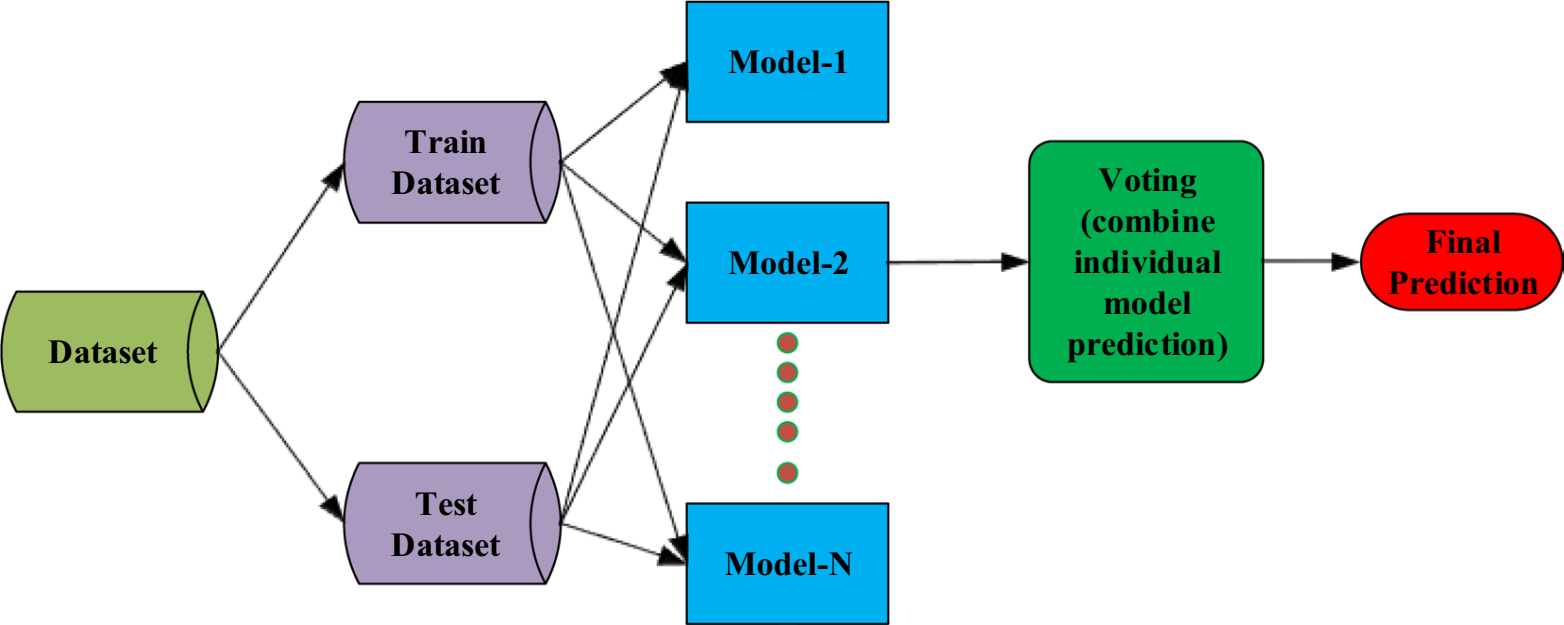
Illustration of AdaBoost method.

AdaBoost, developed by Freund and Schapire in 1996^76^, combines weak learners to create a robust forecasting model. It iteratively trains weak classifiers, assigning higher weights to misclassified instances to enhance performance. The underlying principle of AdaBoost revolves around iteratively training weak classifiers and assigning greater weights to misclassified samples in subsequent iterations. This adaptiveness of AdaBoost improved its overall performance. The process begins with assigning equal weights to each training sample, and a weak classifier is trained to predict the target variable^77^. The weak classifier's performance is then evaluated, and the weights of misclassified samples are increased to emphasize their importance in subsequent iterations. As AdaBoost progresses, subsequent weak classifiers are trained with adjusted weights to provide more accurate predictions^78^. Every weak classifier has attributed a weight corresponding to its performance level, and the final model is created by combining the weak classifiers' predictions weighted by their respective weights^79^. The final model gives higher importance to the weak classifiers that performed better during training. The overall process of AdaBoost modeling is illustrated in Fig. 4.

### Optimization algorithms

#### Particle swarm optimization (PSO)

Kennedy and Eberhart^69^ pioneered the development of an optimization approach known as PSO for addressing optimization challenges. The PSO method draws inspiration from the collective behavior of insects or birds. The PSO begins with the initialization of a population of particles, with each particle showing a potential solution to the problem. These particles possess positions within the solution space and velocities that control their movement. Throughout the optimization process, particles dynamically adjust their positions based on their own experiences and the influence of neighboring particles, ultimately converging toward optimal solutions^70^.

#### Firefly algorithm (FFA)

The FFA is another nature-inspired optimization technique developed to tackle optimization tasks. Inspired by the flashing behavior of fireflies, FFA mimics the attractiveness of fireflies to optimize solutions^71^. The FFA initializes a population of fireflies, each representing a potential solution in the search space. These fireflies exhibit attractiveness, which diminishes with distance, akin to the light intensity of fireflies. During the iterative process, fireflies move towards brighter (i.e., better) solutions in the search space, guided by their attractiveness and the brightness of neighboring fireflies. Through successive iterations, FFA efficiently explores and converges towards optimal solutions in complex optimization tasks^72^.

#### Grey wolf optimizer (GWO)

The GWO is a metaheuristic optimization approach inspired by the social hierarchy and hunting behavior of grey wolves^73^. Developed as a nature-inspired algorithm, GWO effectively tackles optimization problems by mimicking the social interactions and hunting strategies of wolf packs. The GWO initializes a population of grey wolves, with each wolf representing a potential solution in the search space. These wolves are organized into a hierarchical structure, with alpha, beta, delta, and omega wolves representing the pack's leadership. Through the exploration and exploitation phases, the wolves collaborate to adapt and converge toward optimal solutions, making GWO a robust and efficient optimization tool for various real-world problems^74^.

## Research methodology

### Modeling dataset

This research conducted a thorough data collection process, compiling a comprehensive dataset from 28 reputable experimental studies^21,23,75–100^ (Supplementary Table S1–3: Supplementary materials). The model's training process excluded data points that deviated more than 20% from the universal pattern. The acquired dataset consists of 397 compressive strength (CS) records, 346 split tensile strength (STS) records, and 146 elastic modulus (E) records. The dataset consisted of cylindrical and cubic concrete samples without any additive materials. As the variations in specimen sizes and shapes affecting strength, the strength values were transformed to a cube with dimensions of 100 mm using transformation factors suggested by Abellán-García^101^. This transformation accounts for variations in specimen shapes and dimensions observed across published experimental works. Additionally, all the testing samples were subjected to air-curing conditions, as reported. A total of seven input variables were chosen, which included the waste foundry sand to cement ratio (WFS/C), waste foundry sand to the fine aggregate ratio (WFS/FA), fine aggregate to the total aggregate ratio (FA/TA), water to cement ratio (W/C), coarse aggregate to cement ratio (CA/C), 1000 superplasticizer to cement ratio (1000SP/C), and age. The input variables were chosen per previous studies' recommendations^50,51^.

Table 1 presents the statistics of the collected dataset. The CS ranges from 11.4 to 53.8 MPa, E ranges from 18.4 to 46.6 GPa, and STS ranges from 1.7 to 4.9 MPa. The standard deviation (SD) measures the dispersion of the data from the average value. A higher SD shows a higher variability, while a lower value indicates that the data records are closer to the mean. Skewness and kurtosis offer insights into the distribution's shape and symmetry. The suggested range for kurtosis is − 10 to + 10, while for skewness, the range is from − 3 to + 3^102,103^. It can be noticed that skewness and kurtosis for all variables fall within the recommended range.

**Table 1:** Descriptive statistics of collected dataset.

Statistics	WFS/C	W/C	CA/C	FA/TA	WFS/FA	1000SP/C	Age	Output
CS (MPa) (number of data: 397)								
Mean	0.322	0.472	2.989	0.290	0.317	2.600	57.617	32.773
Standard Error	0.0148	0.002	0.026	0.004	0.0203	0.249	4.485	0.421
Mode	0	0.5	2.99	0.33	0	0	28	19.931
Median	0.25	0.47	2.99	0.29	0.18	0	28	32.283
Sample variance	0.087	0.001	0.283	0.008	0.163	24.620	7986.888	70.430
Standard deviation	0.295	0.0418	0.532	0.090	0.404	4.961	89.369	8.392
Skewness	0.971	− 0.071	− 0.680	1.646	2.300	2.201	2.696	0.116
Kurtosis	0.295	− 1.167	3.602	6.450	6.428	3.685	6.454	− 0.484
Minimum	0	0.4	0.93	0.1	0	0	1	11.435
Range	1.2	0.16	3.27	0.59	2.33	17.8	364	42.378
Maximum	1.2	0.56	4.2	0.69	2.33	17.8	365	53.813
E (GPa) (number of data: 146)								
Mean	0.291	0.455	2.689	0.329	0.238	2.973	64.369	30.455
Standard error	0.024	0.003	0.042	0.007	0.026	0.415	8.467	0.557
Mode	0	0.5	2.53	0.3	0	0	28	30
Median	0.21	0.48	2.97	0.31	0.18	0	28	30
Sample variance	0.087	0.002	0.268	0.0078	0.103	25.219	10,468.08	45.372
Standard deviation	0.296	0.047	0.518	0.088	0.321	5.021	102.313	6.735
Skewness	1.310	0.0353	− 0.744	0.524	2.143	1.864	2.407	0.564
Kurtosis	1.442	− 1.549	− 0.513	− 0.081	3.645	2.074	4.480	− 0.467
Minimum	0	0.4	1.49	0.1	0	0	3	18.4
Range	1.28	0.15	1.81	0.4	2.33	15.86	362	28.25
Maximum	1.28	0.55	3.3	0.5	2.33	15.86	365	46.65
STS (MPa) (number of data: 242)								
Mean M Mean	0.365	0.468	2.840	0.314	0.317	2.616	53.512	3.204
Standard error	0.020	0.003	0.036	0.006	0.024	0.325	5.591	0.050
Mode	0	0.5	2.53	0.33	0	0	28	2.66
Median	0.3	0.47	2.93	0.31	0.25	0	28	3.15
Sample variance	0.101	0.002	0.322	0.009	0.144	25.576	7565.421	0.613
Standard deviation	0.317	0.047	0.568	0.095	0.380	5.057	86.979	0.783
Skewness	0.716	0.077	− 0.581	0.828	2.221	2.141	1.906	0.236
Kurtosis	− 0.250	− 1.269	0.965	2.088	6.788	3.377	7.605	− 0.752
Minimum	0	0.4	0.93	0.1	0	0	3	1.69
Range	1.28	0.16	3.27	0.59	2.33	17.8	362	3.21
Maximum	1.28	0.56	4.2	0.69	2.33	17.8	365	4.9

**Figure 5:**
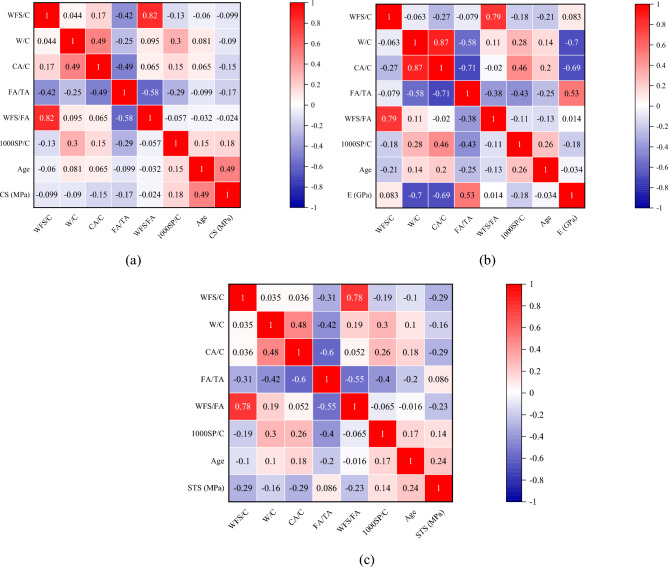
Pearson’s correlation matrix: (**a**) CS, (**b**) E, (**c**) STS.

**Figure 6:**
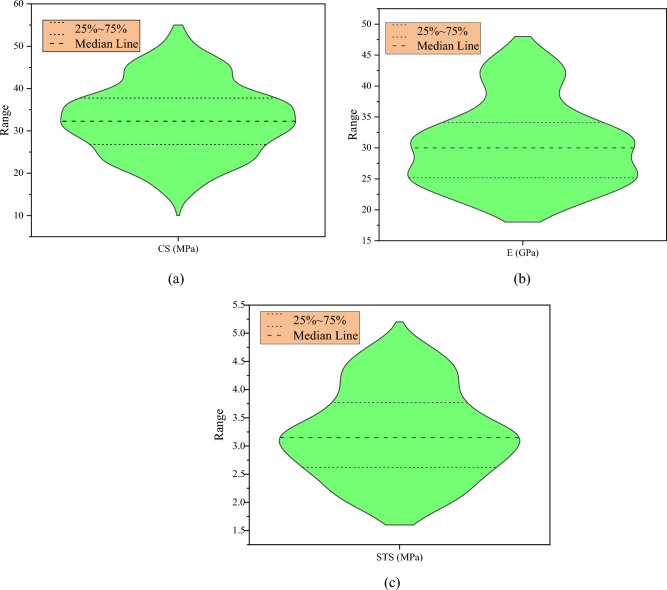
Violin plot showing the distribution of data: (**a**) CS, (**b**) E, (**c**) STS.

**Figure 7:**
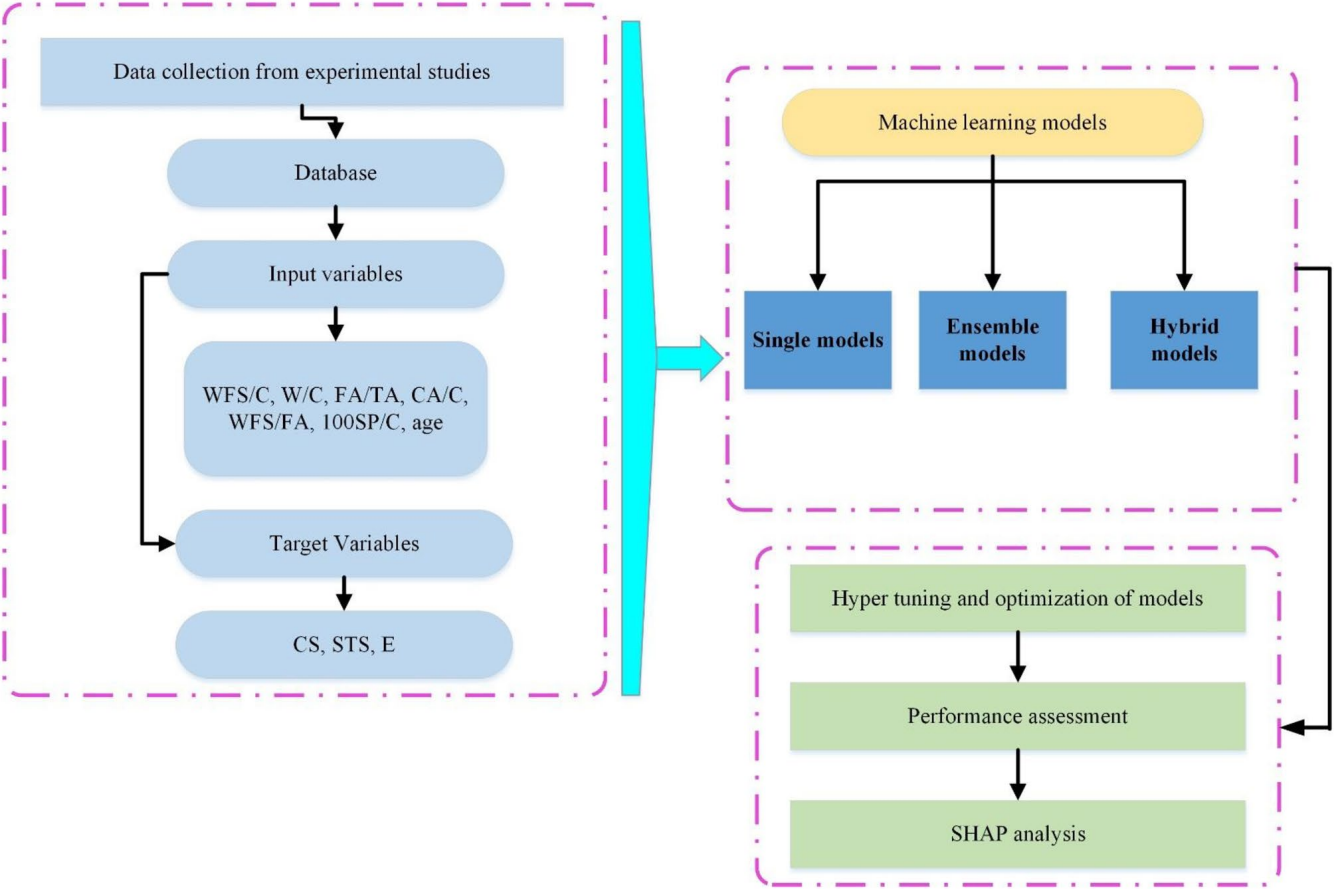
The methodology followed in the present study.

Moreover, there are risks of multicollinearity in prediction models. Multicollinearity refers to the high correlation between two predictors in a regression model. It can be an issue in machine learning as it makes it hard to interpret the model and creates an overfitting problem. The presence of high correlations makes it challenging to determine the unique contribution of each predictor to the outcome variable. Pearson correlation (r) measures the linear correlation between two variables^104^. It is often used to identify multicollinearity in a regression model. If the correlation coefficient between two predictors is high, it indicates a strong linear relationship between them, which can lead to multicollinearity. Generally, for a valid ML model, the r value between two predictors (explanatory variables) must be less than 0.8^105,106^. It can be seen in Fig. 5 that mostly the r value between input variables is lower than 0.8, indicating that there are rare chances of multicollinearity and interdependency. In addition, compressive strength and split tensile strength correlate more (r = 0.49, 0.24, respectively) with age, while elastic modulus correlates more with FA/TA (r = 0.53). Furthermore, the distribution of output parameters is provided in Fig. 6. Figure 7 illustrates the methodology followed in the current study.

### Model development

The collected dataset was split into three subsets: training (70%), validation (15%), and testing (15%). This partitioning strategy ensures that the model is trained on a substantial portion of the data while also having separate subsets for fine-tuning hyperparameters and evaluating performance. The training set, comprising 70% of the data, serves as the primary source for model learning, allowing it to capture underlying patterns and relationships. The validation set, representing 15% of the data, is utilized during the training process to assess the model's performance on unseen data and guide adjustments to hyperparameters, preventing overfitting to the training set. Finally, the testing set, also encompassing 15% of the data, serves as an independent benchmark to evaluate the model's generalization ability accurately. This rigorous partitioning scheme facilitates robust model development and ensures reliable performance estimation on new, unseen data.

Optimizing hyperparameters is crucial for developing ML models effectively, as it helps to make accurate prediction models without overfitting or underfitting. In this study, the grid search approach was employed to find the best hyperparameters for DT and SVR models. During hyperparameter tuning, some data (the testing set) was kept hidden to enhance prediction performance and prevent overfitting. Grid search evaluates every possible combination to determine the best hyperparameter values, ensuring optimal model performance. The optimized hyperparameter values for DT and AR models are given in Table 2. Along with DT and AR models, three hybrid models by optimization of SVR with three metaheuristic methods such as FFA, PSO, and GWO were also developed. For the hybrid models, the key parameters for SVR are C (penalty factor), ε (margin of tolerance), and γ (kernel coefficient), with ranges set at (0.01,100), (0.01, 1.0), and (1.0, 10), respectively. Then, three metaheuristic algorithms, namely GWO, FFA, and PSO are employed, to improve the SVR approach ability to predict the strength properties of WFS-based concrete and to decrease parameter search time. The parameters for GWO, FA, and PSO are configured according to the specifications outlined in Table 2.

**Table 2:** Parameters setup of the developed models.

Method	Parameter	Range	Optimized value
DT	Max depth	Integer: 1–100	10
	Min samples split	Integer/float: 2–10	5
	Min samples leaf	Integer/float: 1–5	1
	Max features	Auto, sqrt, log2	Auto
AdaBoost	Base estimator	Any estimator	DecisionTree
	n_estimators	Integer: 50–200	100
	learning_rate	Float: 0.01–1.0	0.1
SVR	Kernel	Linear, Poly, RBF	RBF
	Regularization Parameter (C)	Float: 0.1–100	1.0
	Kernel Coefficient (γ)	Float: 0.1–10	0.1
	Epsilon (ε)	Float: 0.01–1.0	0.1
	Max Iterations (max_iter)	Integer: 100–1000	500
FFA	α	Float: 0.1–1.0	0.5
	rand	Float: 0.0–1.0	0.3
	β0	Float: 0.1–1.0	0.8
	γ	Float: 0.1–1.0	0.2
PSO	Vmax	Float: 0.1–10	2.0
	Wmin	Float: 0.1–1.0	0.2
	Wmax	Float: 1.0–5.0	3.0
	c1	Float: 0.1–2.0	1.0
	c2	Float: 0.1–2.0	1.5
GWO	Alpha (α)	Float: 0.1–1.0	0.7
	Beta (β)	Float: 0.1–1.0	0.5
	Delta (δ)	Float: 0.1–1.0	0.3

### Evaluation of model performance

In ML modeling, it is essential to assess the performance of a model to determine its accuracy and effectiveness. Various evaluation metrics are employed to gauge the model's performance. When dealing with regression tasks, several commonly used metrics include correlation coefficient (R), root mean squared error (RMSE), mean absolute error (MAE), relative root mean squared error (RRMSE), performance index (PI), and relative squared error (RSE). These metrics serve as reliable indicators to determine the accuracy and predictive capabilities of the model. The expressions of these metrics are given as Eqs. (1)–(5).

1 $$\text{RMSE }= \sqrt{\frac{\sum_{\text{i=1}}^{\text{n}}\text{(}{\text{ei-mi)}}^{2}}{\text{n}}}$$

2 $$\text{MAE} = \frac{\sum_{\text{i=1}}^{\text{n}}\text{|ei-mi|}}{\text{n}}$$

3 $$\text{RRMSE }= \frac{1}{|\overline{e} |} \sqrt{\frac{\sum_{\text{i=1}}^{\text{n}}{\text{(ei-mi)}}^{2}}{\text{n}}}$$

4 $${\text{R}}\; = \;\frac{{\mathop \sum \nolimits_{{\text{i}} = 1}^{\text{n}} ({\text{ei}} - \bar e{\text{i}})({\text{mi}} - \;\bar m{\text{i}})\;}}{{\sqrt {\mathop \sum \nolimits_{{\text{i}} = 1}^{\text{n}} {{({\text{ei}} - \;\bar e{\text{i}})}^2}\mathop \sum \nolimits_{{\text{i}} = 1}^{\text{n}} {{({\text{mi}} - \bar m{\text{i}})}^2}} }}$$

5 $$\text{PI }= \frac{\text{RRMSE}}{\text{1+R}}$$

where "ei" and "mi" denote the actual and estimated values, respectively, while "ēi" and "${\bar {{\text{m}}}} {\text{i}}$" represents the mean of actual and estimated values.

R metric determines the correlation between the model and actual values. An R-value closer to 1 (R > 0.8) is considered to be an excellent accuracy of the model. However, correlation value alone cannot be utilized as a sole measure of performance accuracy since it only determines the linear relationship between two variables. While correlation is a useful metric for understanding the direction of the relationship, it does not capture a model's overall accuracy or predictive power. Therefore, it is crucial to consider other performance measures to gauge the model's accuracy properly. RMSE is a metric that quantifies the average disparity between the predicted values from a statistical model and the corresponding actual values. RMSE is the standard deviation of residuals that deviates from the observed records. The residuals essentially indicate the extent to which the model's estimations deviate from the observed values. Both MAE and RMSE values closer to 0 indicate a better model performance. The PI metric is also an excellent measure to gauge the model accuracy as it considers RRMSE and R simultaneously. Its value lower than 0.2 represents a better performance of the model.

Model overfitting happens when a machine learning model shows excellent performance on the data it was trained on but struggles to perform well on new, unseen data. This occurs because the model learns noise and relies on random or irrelevant patterns present in the training data, which do not apply to new data, resulting in poor predictions^107^. Accordingly, the objective function (OBF) metric (Eq. 6) assesses model overfitting. A value of OBF less than 0.2 indicates that the issue of model overfitting has been resolved.

6 $$\text{OBF }= (\frac{{\text{n}}_{\text{T}}-{\text{n}}_{\text{v}}}{\text{n}}){\text{PI}}_{\text{T}} + 2(\frac{{\text{n}}_{\text{v}}}{\text{n}}){\text{PI}}_{\text{V}}$$

The subscripts "T" and "V" represent the training and validation, respectively, while "n" denotes the number of datasets. Furthermore, the criteria for external validation are provided in Table 3.

**Table 3:** External validation criteria.

S.No.	Equation	Range	Suggested by
1	${\text{k}} = \frac{{\sum\nolimits_{{\text{i}} = 1}^{\text{n}} {({\text{ei }} \times {\text{mi}})} }}{{{\text{e}}{{\text{i}}^2}}}$	$\text{0.85 < k < 1.15}$	^108^
2	${k^{\prime}=}\frac{\sum_{\text{i=1}}^{\text{n}}({\rm ei} \times {\rm mi})}{{\text{mi}}^{2}}$	${0.85 < k^{\prime} < 1.15}$	^108^
3	${\text{m}}=\frac{{\text{R}}^{2} - {\text{ R}}_{o}^{2}}{{\text{R}}^{2}}$	m < 0.1	^109^
4	${\text{R}}_{\text{m}}\text{=}{\text{R}}^{2} \times \text{(1-}\sqrt{\text{|}{\text{R}}^{2 } - {\text{R}}_{o}^{2}}|)$	R_m_ > 0.5	^110^
	where		
	${\text{R}}^{2}\text{=1-} \frac{{\sum }_{\text{i=1}}^{\text{n}}{\text{(mi-} {\text{e}}_{\text{i}}^{o}\text{)}}^{2}}{{{\sum }_{\text{i=1}}^{\text{n}}\text{(mi-} {\text{m}}_{\text{i}}^{\text{o}}\text{)}}^{2}}$	${\text{R}}^{2}{\cong }{1}$	
	${\text{R}}_{o}^{2}\text{=1- }\frac{{\sum }_{\text{i=1}}^{\text{n}}{\text{(ei- }{\text{m}}_{\text{i}}^{o}\text{)}}^{2}}{{{\sum }_{\text{i=1}}^{\text{n}}\text{(ei- }{\text{e}}_{\text{i}}^{\text{o}}\text{)}}^{2}}$	${\text{R}}_{o}^{2}{\cong }{1}$	
	$${\text{e}}_{\text{i}}^{\text{o}} = {\text{k}} \times {{\text{m}}_{\text{i}}},\;\;{\text{m}}_{\text{i}}^{\text{o}} = {\text{k}} \times {{\text{e}}_{\text{i}}}$$		

### Model interpretation

Previous research on concrete characteristics using machine learning has often focused on achieving higher accuracy while neglecting the importance of model interpretation. However, model interpretation is a crucial aspect that should not be overlooked. Understanding how the model arrives at its predictions can provide valuable insights and enhance the trustworthiness and applicability of the results. Accordingly, the SHAP method^111^ is employed in the current study to interpret the model prediction results. SHAP analysis is a versatile approach that can be applied to any machine learning model. It leverages the principles of game theory to provide explanations for model outputs. By utilizing Shapley values derived from coalitional game theory, SHAP assigns credit to each feature's contribution towards the prediction. It ensures an excellent distribution of the "payout" (i.e., the prediction) among the features based on their individual or grouped values^112,113^. SHAP analysis is a powerful and flexible technique for interpreting machine learning models, as it is model-agnostic, consistent, and capable of handling complex behaviors. It is particularly valuable in understanding model functioning, identifying important features, and explaining prediction outcomes.

## Results and discussion

### Performance evaluation of the models

#### Regression analysis

This section aimed to evaluate the effectiveness of the suggested models through a regression analysis, specifically by assessing the slope of the line derived from plotting experimental results along the x-axis against predicted results along the y-axis. Such a type of assessment of the ML models has been extensively employed in previous studies^12,114–116^. For instance, while investigating the compressive strength of concrete incorporated with rice husk ash, Iqtidar et al.^117^ found the regression line slope equal to 0.99 for validation and training sets. Generally, a regression slope (RS) higher than 0.8 and closer to 1 is considered best for an optimal predictive model^118^.

**Figure 8:**
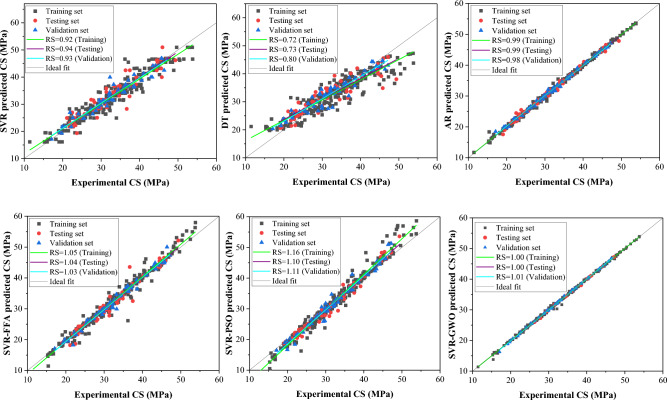
Regression slopes analysis of the developed model for compressive strength.

**Figure 9:**
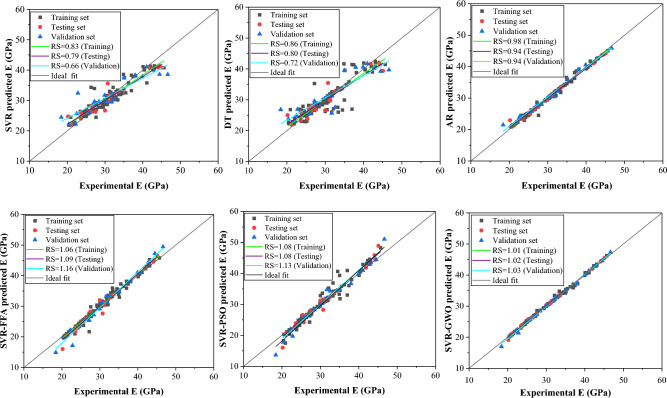
Regression slopes analysis of the developed model for elastic modulus.

**Figure 10:**
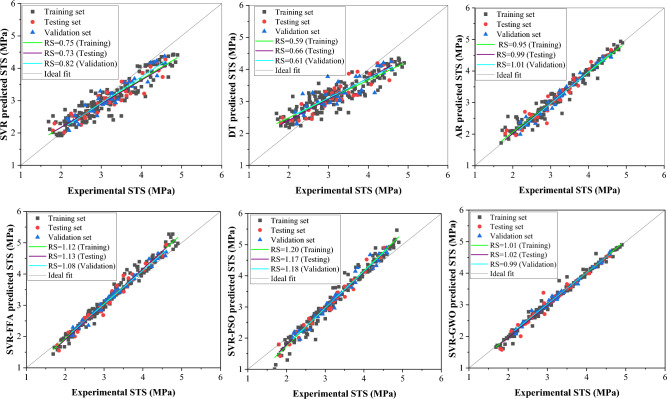
Regression slopes analysis of the developed model for split tensile strength.

Figure 8 displays regression plots illustrating the regression slope performance of the CS models. The SVR model exhibited a good regression slope compared to DT, while the latter showed a lower regression slope falling below the recommended threshold of 0.80, indicating poor performance for CS estimation. Conversely, the ensemble model (AR) demonstrated a higher regression slope, alongside SVR-based hybrid models showing excellent performance. Overall, AR and SVR-GWO models exhibited superior accuracy in RS analysis, suggesting their potential for estimating compressive strength of WFSC. Furthermore, Fig. 9 depicts regression plots demonstrating the performance of the E models. Standalone models displayed subpar regression slopes for estimating elastic modulus of WFSC, while the AR-based ensemble model showed improved performance. SVR-based hybrid models exhibited excellent regression slope performance, indicating their capability to accurately capture WFSC intricacies and provide accurate predictions. Notably, the SVR-GWO model demonstrated greater prediction accuracy in terms of regression slope in estimating E, with RS values for training, validation, and testing approaching 1. Moreover, the DT and SVR models also showed poor regression slope performance in estimating the split tensile strength of WFSC, as shown in Fig. 10. The ensemble and SVR-based hybrid models showed improved prediction accuracy in terms of regression slope. Notably, the AR and SVR-GWO models exhibited excellent regression slopes compared to other models, indicating their potential to accurately estimate the STS of WFSC.

#### Error analysis

**Figure 11:**
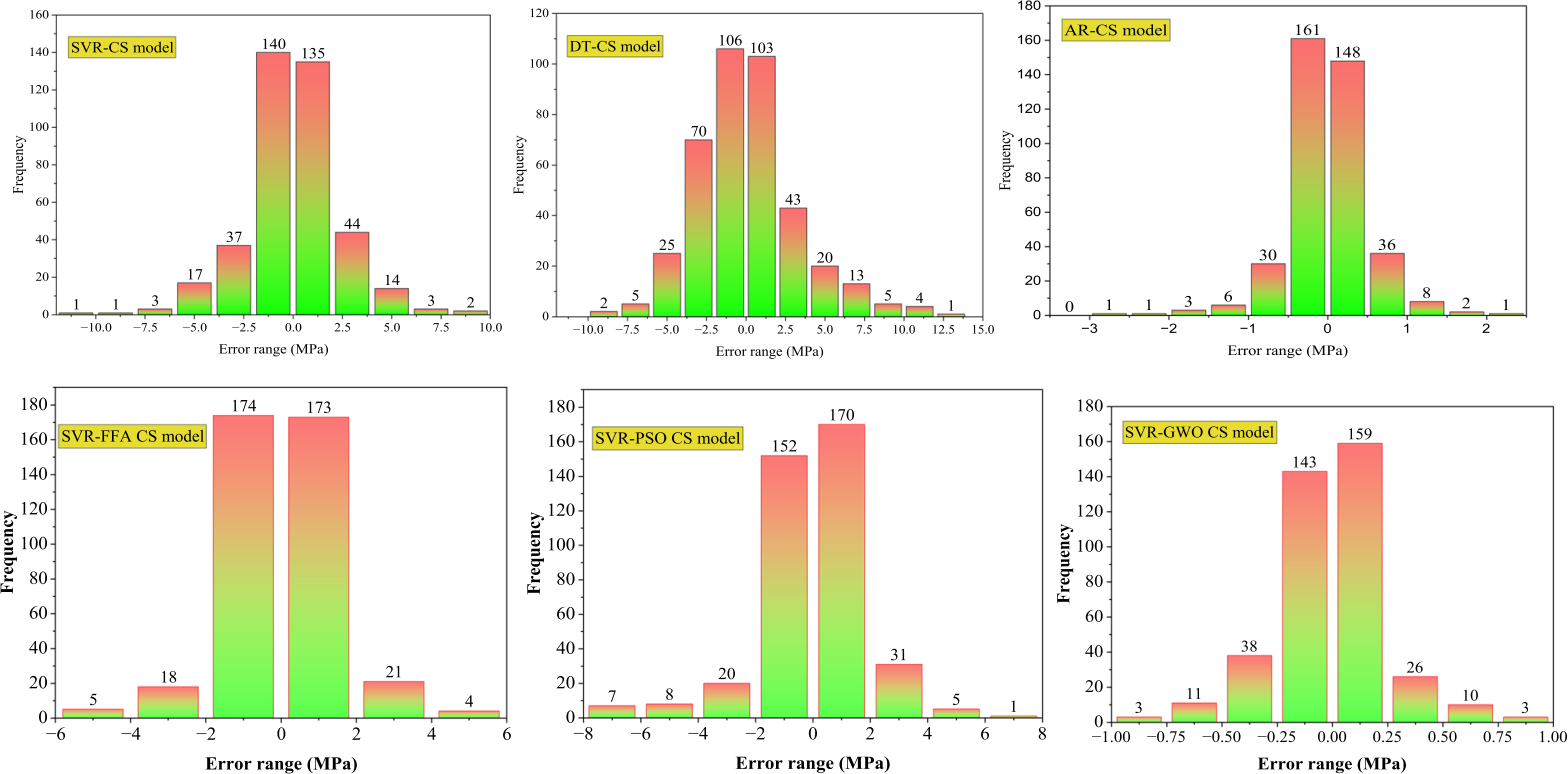
Error histograms of the developed models for CS.

**Figure 12:**
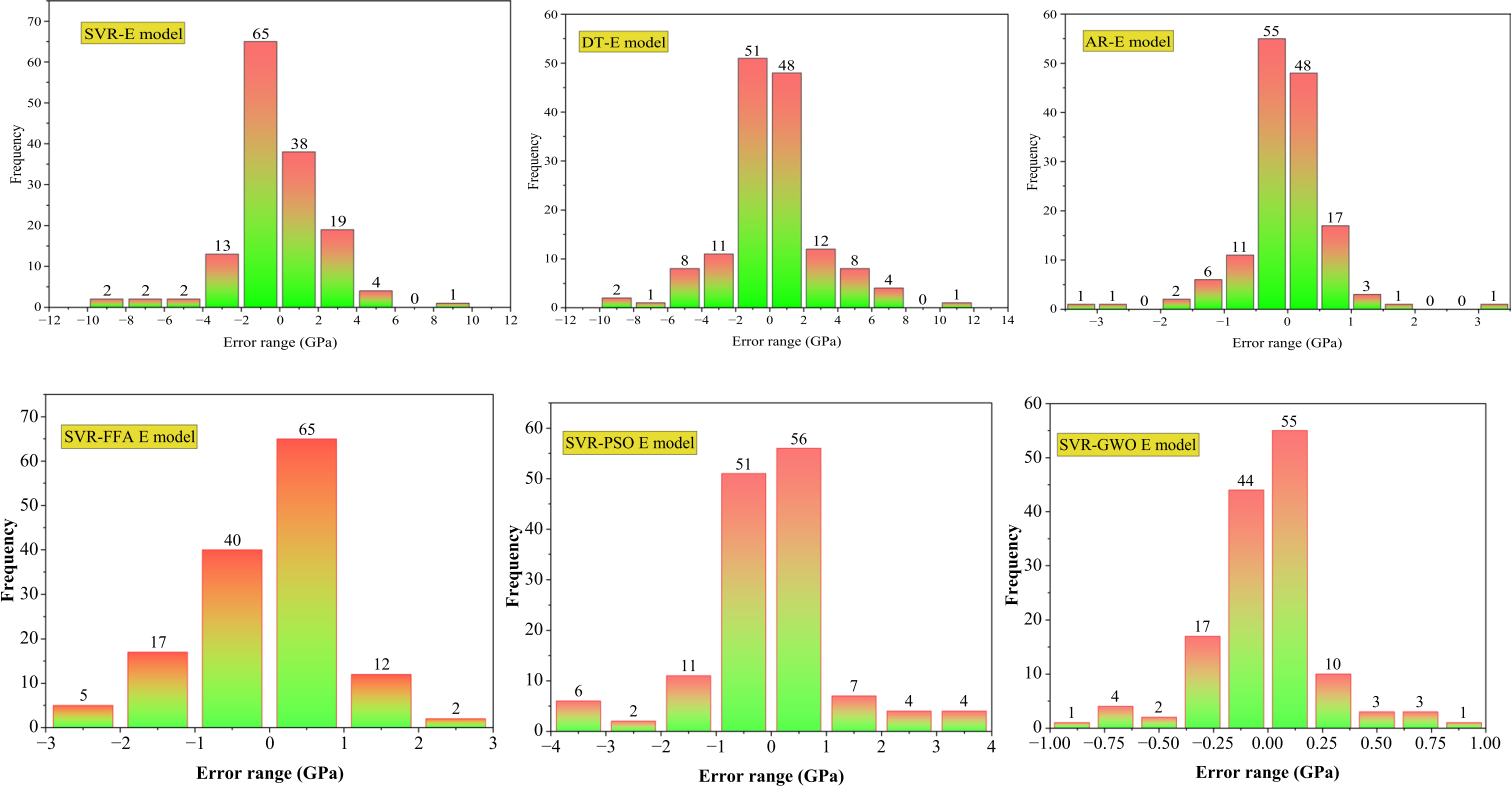
Error histograms of the developed models for E.

**Figure 13:**
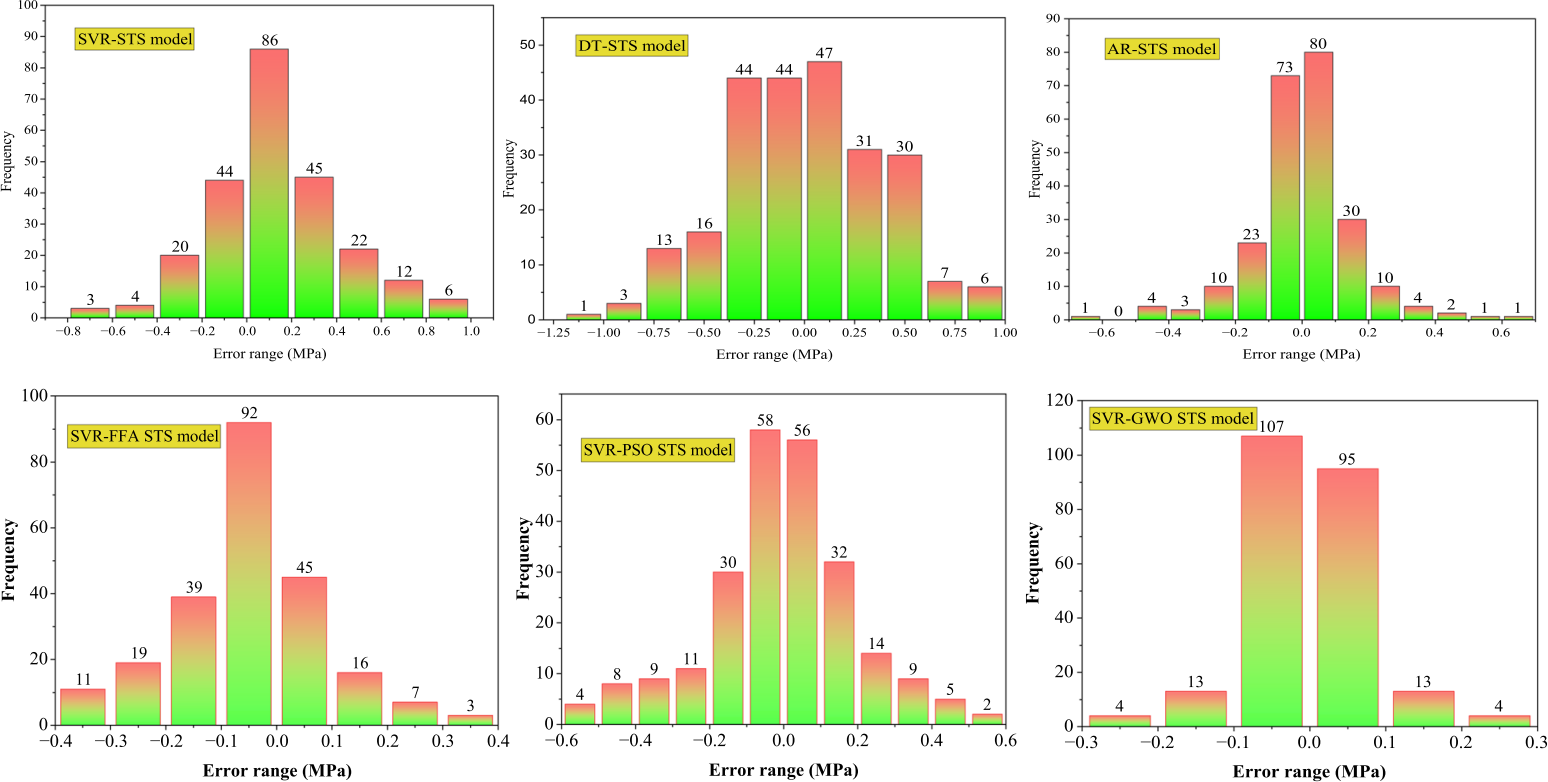
Error histograms of the developed models for STS.

Figure 11 shows the error histograms of the models established for CS. The error analysis of the SVR model for CS showed that 97.5% of errors lie in the range of ± 5 MPa. Similarly, the DT model also exhibits moderate precision, with 92.5% errors falling in the same range of ± 5 MPa. However, the AR-CS model outperforms both, delivering exceptional precision with 94.5% confined in the range of ± 1 MPa. The SVR-FFA and SVR-PSO models exhibited 87.40% and 81.10% error in error range of ± 2 MPa, respectively. The SVR-GWO model demonstrated improved accuracy with 76.07% error fall in the range of ± 0.25 MPa. Furthermore, Fig. 12 displays the error histograms of the models established for E. The SVR model for E provided 92.5% errors within the range of ± 4 GPa, and DT observed 83.5% errors within the range of ± 4 GPa. The AR model provided excellent performance with 89.7% errors in the range of ± 1 GPa. The SVR-FFA and SVR-PSO models for E showed 91.71% and 73.28% of the error in the range of ± 1 GPa, respectively. In contrast, the SVR-GWO exhibited improved precision for estimating E with 86.30% error in the range of ± 0.25 GPa. Moreover, Fig. 13 shows the error histograms of the established models for STS. The SVR model provided 80.6% errors in the range of ± 0.4 MPa, while the DT model evidenced 81.1% errors in the range of 0.5 MPa. Moreover, the AR model exhibited 85.1% errors in the range of ± 0.2 MPa. The SVR-FFA and SVR-PSO models exhibited 79.33% and 72.72% of the predictions, respectively, within a range of ± 2 MPa. However, the SVR-GWO model showed the highest accuracy with error of 83.47% of the predictions within error range of ± 0.1 MPa. Overall, the SVR-GWO models exhibited less errors compared to other established models in estimating the CS, STS, and E of waste foundry sand concrete.

#### Statistical assessment of the models

Along with regression and error analysis, performance metrics were employed to determine the accuracy and performance of the developed models, as provided in Table 4. For predicting the CS of WFSC, AR, and SVR-based hybrid models provided higher accuracy (R) and lower error (MAE, RMSE) values. Notably, for CS prediction, the SVR-GWO model provided better accuracy, while the DT model exhibited lower accuracy. Similarly, the SVR-GWO model exhibited the highest accuracy in estimating elastic modulus with R-value of 0.999 for three subsets. In addition, the SVR-GWO model showed the lowest RMSE and MAE values for estimating E of WFSC. Furthermore, it can be noticed that the SVR-GWO model for STS also provided better accuracy compared to other developed prediction models. The SVR-GWO model for STS exhibited R-values of 0.994, 0.985, and 0.996 for training, validation, and testing, respectively. Moreover, the OBF is lower than 0.2 for all established models, effectively addressing the issue of model overfitting. Overall, the error value is minimal, and R values are higher than the recommended threshold (0.8), illustrating that the developed models can precisely predict the strength characteristics of waste foundry sand concrete.

**Table 4:** Statistical metrics values for the developed models.

Model	Subset	RMSE	MAE	RRMSE	R	PI	OBF
CS models							
SVR	Train	2.435	1.753	0.074	0.955	0.038	0.036
	Valid	2.216	1.597	0.067	0.966	0.034	
	Test	2.153	1.632	0.069	0.962	0.039	
DTd DT	Train	3.322	2.503	0.101	0.926	0.052	0.052
	Valid	3.361	2.567	0.102	0.930	0.053	
	Test	3.2822	2.383	0.098	0.943	0.051	
AR	Train	0.534	0.364	0.016	0.998	0.008	0.007
	Valid	0.449	0.326	0.014	0.999	0.007	
	Test	0.435	0.316	0.013	0.999	0.005	
SVR-FFA	Train	1.302	0.832	0.040	0.991	0.020	0.018
	Valid	1.510	1.010	0.046	0.987	0.023	
	Test	2.114	1.170	0.065	0.976	0.033	
SVR-PSO	Train	2.256	1.400	0.069	0.985	0.035	0.023
	Valid	1.867	1.189	0.057	0.988	0.029	
	Test	1.932	1.247	0.059	0.988	0.030	
SVR-GWO	Train	0.287	0.179	0.009	0.999	0.004	0.003
	Valid	0.326	0.176	0.010	0.999	0.005	
	Test	0.303	0.167	0.009	0.999	0.005	
E models							
SVR	Train	2.145	1.646	0.005	0.947	0.002	0.026
	Valid	2.457	1.762	0.080	0.925	0.041	
	Test	2.132	1.542	0.004	0.953	0.002	
DT	Train	2.332	1.938	0.011	0.907	0.006	0.029
	Valid	2.568	1.750	0.084	0.911	0.044	
	Test	2.4711	1.854	0.072	0.912	0.017	
AR	Train	0.699	0.471	0.022	0.995	0.011	0.011
	Valid	0.658	0.413	0.021	0.995	0.010	
	Test	0.664	0.421	0.021	0.996	0.010	
SVR-FFA	Train	1.025	0.686	0.034	0.990	0.017	0.017
	Valid	1.227	1.227	1.227	0.998	0.020	
	Test	1.985	1.465	0.065	0.990	0.033	
SVR-PSO	Train	1.443	0.863	0.047	0.981	0.024	0.020
	Valid	1.331	0.840	0.044	0.988	0.022	
	Test	2.085	1.449	0.068	0.987	0.034	
SVR-GWO	Train	0.335	0.196	0.011	0.999	0.006	0.005
	Valid	0.296	0.191	0.010	0.999	0.005	
	Test	0.512	0.294	0.017	0.999	0.008	
STS models							
SVR	Train	0.321	0.248	0.100	0.914	0.052	0.053
	Valid	0.316	0.229	0.098	0.952	0.050	
	Test	0.318	0.241	0.099	0.943	0.061	
DT	Train	0.384	0.308	0.119	0.875	0.063	0.065
	Valid	0.380	0.301	0.118	0.903	0.062	
	Test	0.373	0.298	0.116	0.921	0.059	
AR	Train	0.165	0.111	0.051	0.974	0.026	0.023
	Valid	0.131	0.087	0.041	0.991	0.020	
	Test	0.152	0.110	0.054	0.983	0.024	
SVR-FFA	Train	0.177	0.120	0.055	0.989	0.028	0.017
	Valid	0.170	0.130	0.053	0.988	0.027	
	Test	0.133	0.107	0.042	0.991	0.021	
SVR-PSO	Train	0.234	0.165	0.073	0.986	0.037	0.024
	Valid	0.183	0.137	0.057	0.989	0.029	
	Test	0.192	0.132	0.060	0.986	0.030	
SVR-GWO	Train	0.093	0.053	0.029	0.994	0.015	0.009
	Valid	0.123	0.070	0.038	0.985	0.019	
	Test	0.073	0.049	0.023	0.996	0.011	

In addition, Table 4 shows the external validation parameter values for the developed models. One of the criteria employed for external validation is to ensure that the regression line slopes (k or k′) are close to one, as proposed in previous studies [136]. Another criterion, known as the confirming indicator (Rm), is used to measure a model's predictability introduced by Roy [137]. The requirement is that Rm must exceed 0.5. In addition, the value of parameter m falls below the threshold of 0.1. Table 5 demonstrates that all three models satisfy the criteria for external validation, suggesting that these models are realistic and not simply a correlation between input and output variables.

**Table 5:** External validation of the models.

`	0.85 < k < 1.15	0.85 < k′ < 1.15	R^2^ $\cong {1}$	${\text{R}}_{o}^{2}\cong {1}$	R_m_ > 0.5	m < 0.1
CS						
SVR	0.995	1.101	0.995	0.992	0.926	− 0.005
DT	0.984	1.007	0.998	0.995	0.948	0.003
AR	0.999	1.001	0.999	0.999	0.978	− 0.0005
SVR-FFA	0.987	1.006	0.997	0.994	0.913	− 0.004
SVR-PSO	0.986	1.012	0.995	0.996	0.943	− 0.004
SVR-GWO	0.999	1.000	0.999	0.999	0.989	− 0.0004
E						
SVR	0.993	1.002	0.990	0.999	0.896	− 0.009
DT	0.988	1.004	0.999	0.999	0.946	− 0.00001
AR	0.998	1.001	0.999	0.999	0.999	0.00002
SVR-FFA	0.997	0.987	0.993	0.999	0.933	− 0.00001
SVR-PSO	0.997	0.989	0.994	0.999	0.954	− 0.0008
SVR-GWO	0.999	1.000	1.001	1.001	1.000	0.00001
STS						
SVR	0.946	1.049	0.849	0.861	0.762	− 0.012
DT	0.978	1.009	0.966	0.994	0.803	− 0.029
AR	0.997	1.000	0.997	0.999	0.942	− 0.003
SVR-FFA	0.986	1.004	0.974	0.993	0.892	− 0.005
SVR-PSO	0.985	1.007	0.982	0.992	0.912	− 0.004
SVR-GWO	0.998	1.000	0.999	0.999	0.976	− 0.0002

### Comparison of the developed models

This section aims to compare the accuracy and error levels of the developed models in estimating the characteristics of WFSC. For CS prediction, the SVR-GWO model showcased the most impressive performance with the lowest MAE values among all suggested models. Although the ensemble model (AR) also demonstrated notable accuracy, it slightly lagged behind the hybrid SVR-GWO model. Similar trends were observed for E and STS prediction, where the SVR-GWO model consistently outperformed others. This observation underscores the robustness and efficacy of the SVR-GWO hybrid model for estimating the strength properties of waste foundry sand concrete.

**Figure 14:**
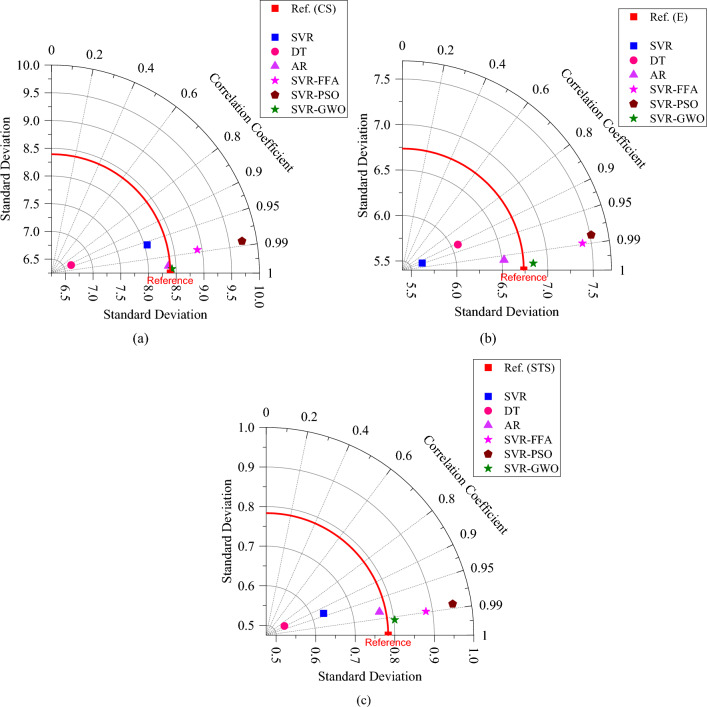
Taylor diagrams: (**a**) CS, (**b**) E, (**c**) STS.

The visual comparison of the developed models is provided in the form of a Taylor diagram (Fig. 14). In Taylor's diagram, each model is represented by a point on the diagram. The distance from the origin to the point reflects the standard deviation, and the radial position represents the correlation. The closer the points are to each other, the higher the similarity and agreement between the models. The reference point (baseline) is provided with R equal to 1 and RMSE equal to 0. It can be observed that in all cases (E, CS, STS), the SVR-GWO and AR model are closer to the reference point, indicating their higher performance accuracy in estimating the strength properties of WFSC.

The comparative analysis of statistical metrics reaffirms the superior accuracy of the SVR-based hybrid and AR models in predicting the strength properties of WFSC. Ensemble models, renowned for their adeptness in handling complex patterns and noise within the data, contribute to the elevated accuracy levels observed. While the ensemble model's enhanced accuracy can be attributed to its utilization of multiple standalone models within an ensemble framework, the introduction of hybrid models such as SVR-GWO, SVR-PSO, and SVR-FFA marks a significant advancement. These hybrid models leverage the strengths of both optimization algorithms and support vector regression (SVR), resulting in improved prediction accuracy. Therefore, it is evident that both hybrid and ensemble models outperform individual or standalone models, indicating a promising accuracy in the predictive modeling of WFSC strength properties.

### SHAP interpretability of the models

The current study offers both global and local interpretations to gain deeper insights into the models' predictions, thus enriching the understanding of the predictive capabilities of the models. Among the three models, the AR model demonstrated excellent accuracy; thus, SVR-GWO prediction results were considered for SHAP analysis.

#### Global interpretation

**Figure 15:**
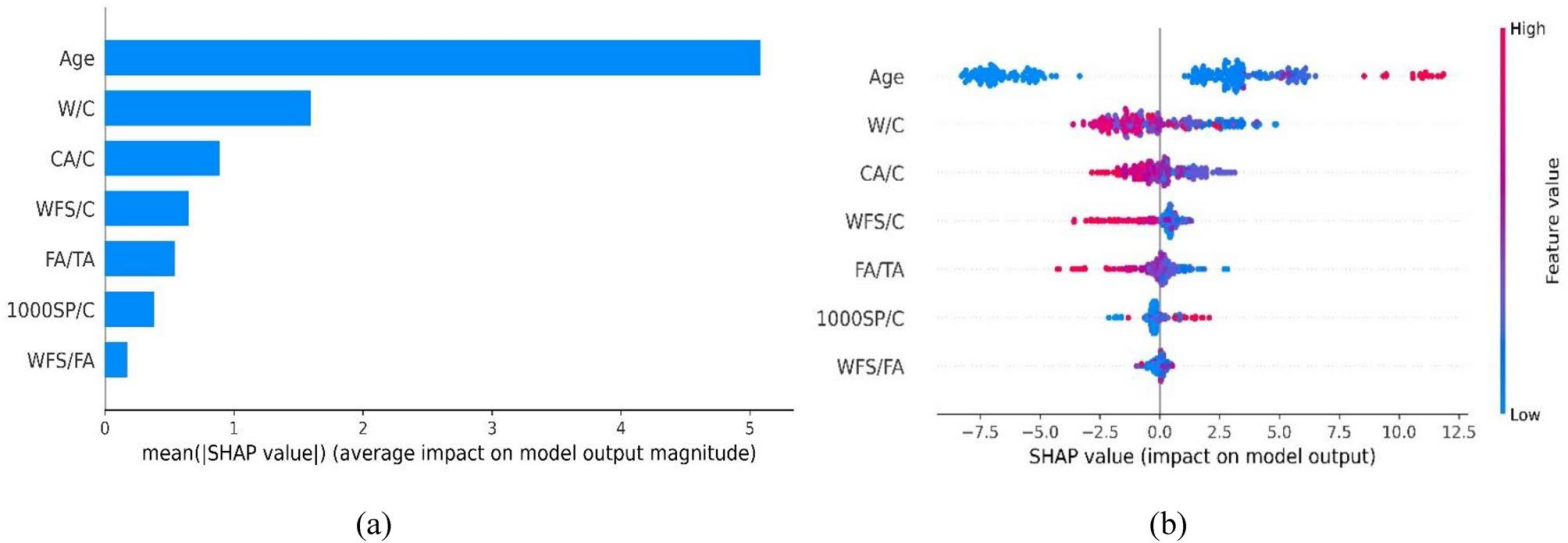
SHAP interpretation for compressive strength model: (**a**) feature importance, (**b**) summary plot.

The global SHAP explanation facilitates a comprehensive understanding of the individual contribution of each input feature towards the output prediction, thereby unraveling the precise influence and impact of each feature on the overall prediction. Age and water-to-cement ratio have a higher contribution in estimating the compressive strength of WFSC. It can also be noticed that CA/C also has considerable significance in predicting CS, as shown in Fig. 15a. Age, W/C, and CA/C combined contribute 82.8% of the total SHAP value for all input features. It can be observed in Fig. 15b that age positively impacts compressive strength, indicating that an increase in age corresponds to an improvement in overall compressive strength. In contrast, higher values of W/C and CA/C negatively influence compressive strength.

**Figure 16:**
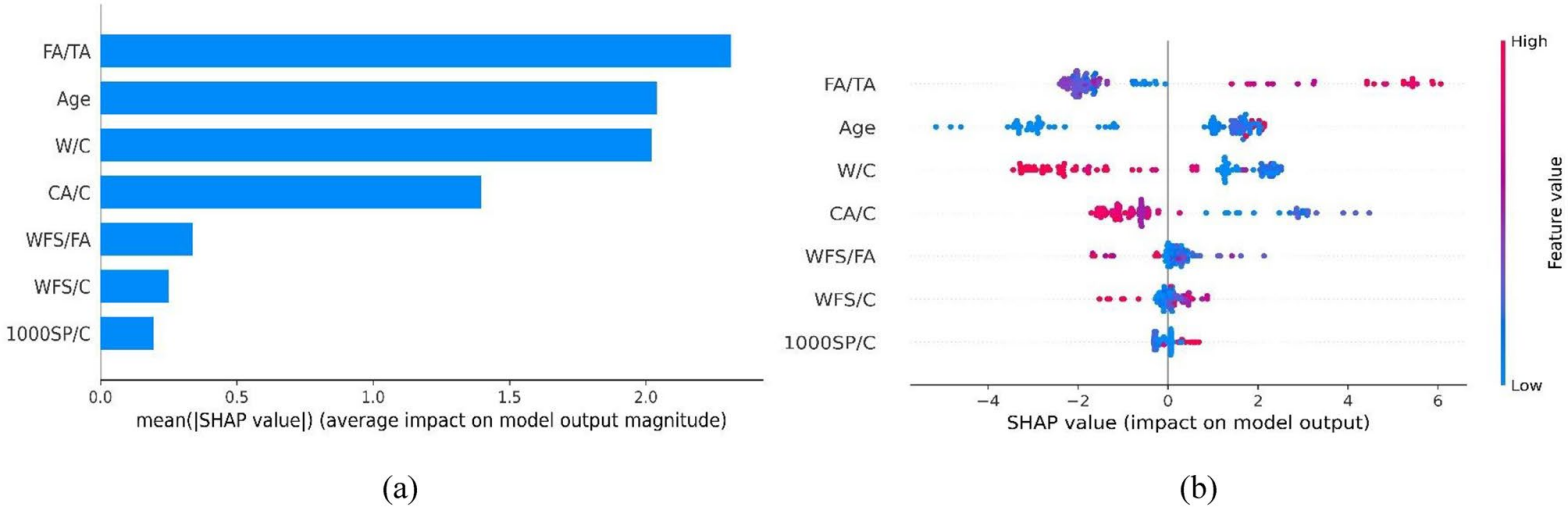
SHAP interpretation for elastic modulus model: (**a**) feature importance, (**b**) summary plot.

Similarly, the SHAP feature importance and the summary plot are provided in Fig. 16. It can be noticed that FA/TA, age, W/C, and CA/C are significantly contributing to the estimation of elastic modulus. However, the rest of the feature contributes very little to the prediction of E, as shown in Fig. 16a. The mean SHAP values of FA/TA, age, W/C, and CA/C are approximately 89.8% of the total SHAP values. The summary plot for elastic modulus is illustrated in Fig. 16b. It can be noticed that the higher values of FA/TA and age enhance the elastic modulus. However, W/C and CA/C negatively influence the elastic modulus of WFSC.

**Figure 17:**
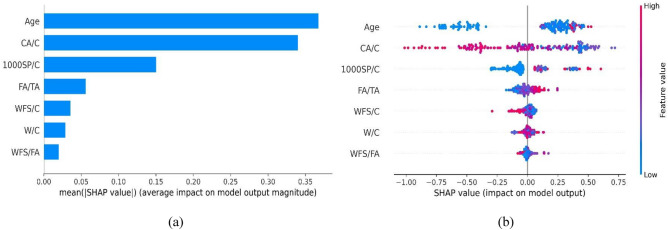
SHAP interpretation for split tensile strength model: (**a**) feature importance, (**b**) summary plot.

Furthermore, age has a more pronounced contribution to the determination of STS, followed by the water-to-cement ratio. Interestingly, the parameter 1000SP/C also exhibits significant importance in predicting STS. The rest of the input features have no significant contribution to STS, as shown in Fig. 17a. The mean SHAP values of age, 100SP/C, and W/C are about 84.5% of the total SHAP value. Increasing the age results in the enhancement of STS, as shown by the red dots on the right side of Fig. 17b. However, an increase in CA/C negatively influences split tensile strength. In addition, an increase in 1000SP/C also enhances the STS.

#### Local interpretation

**Figure 18:**
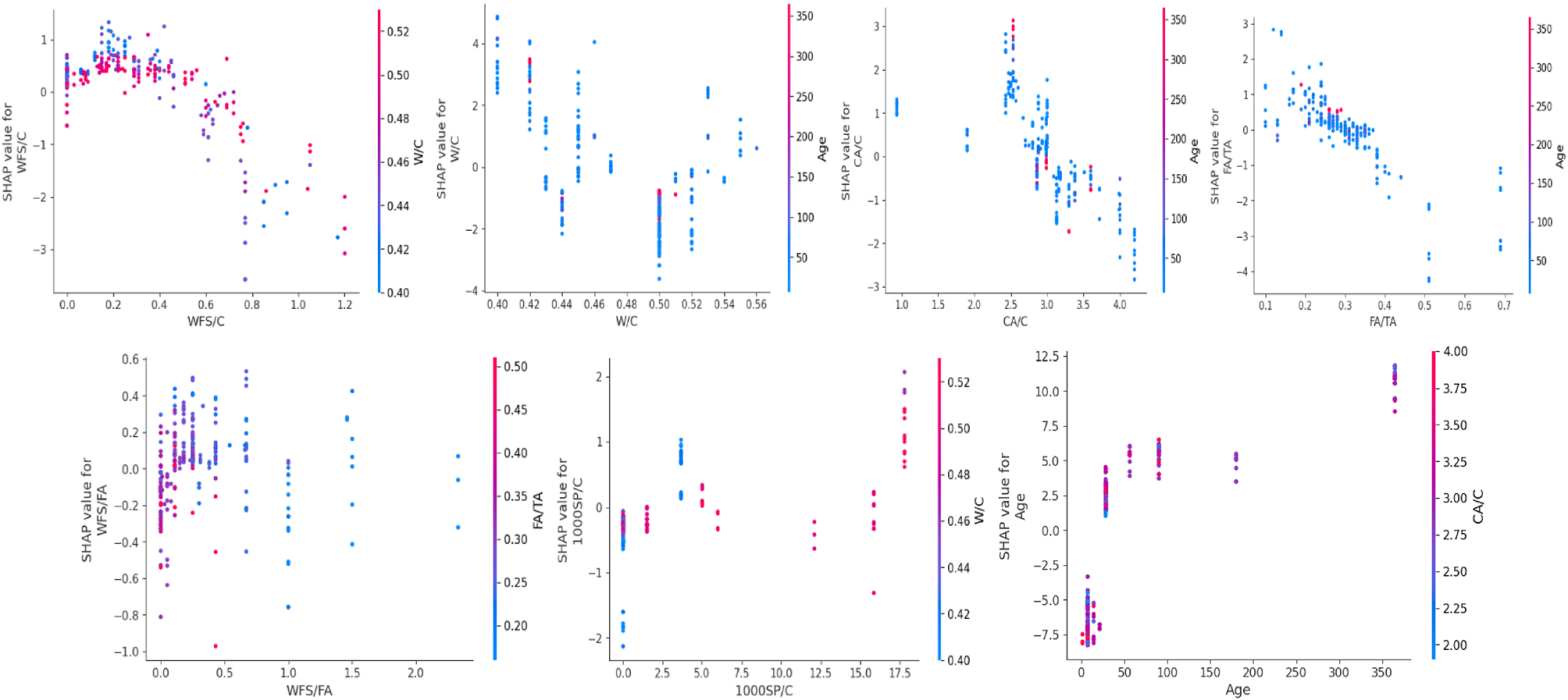
Features interaction plots for compressive strength model.

**Figure 19:**
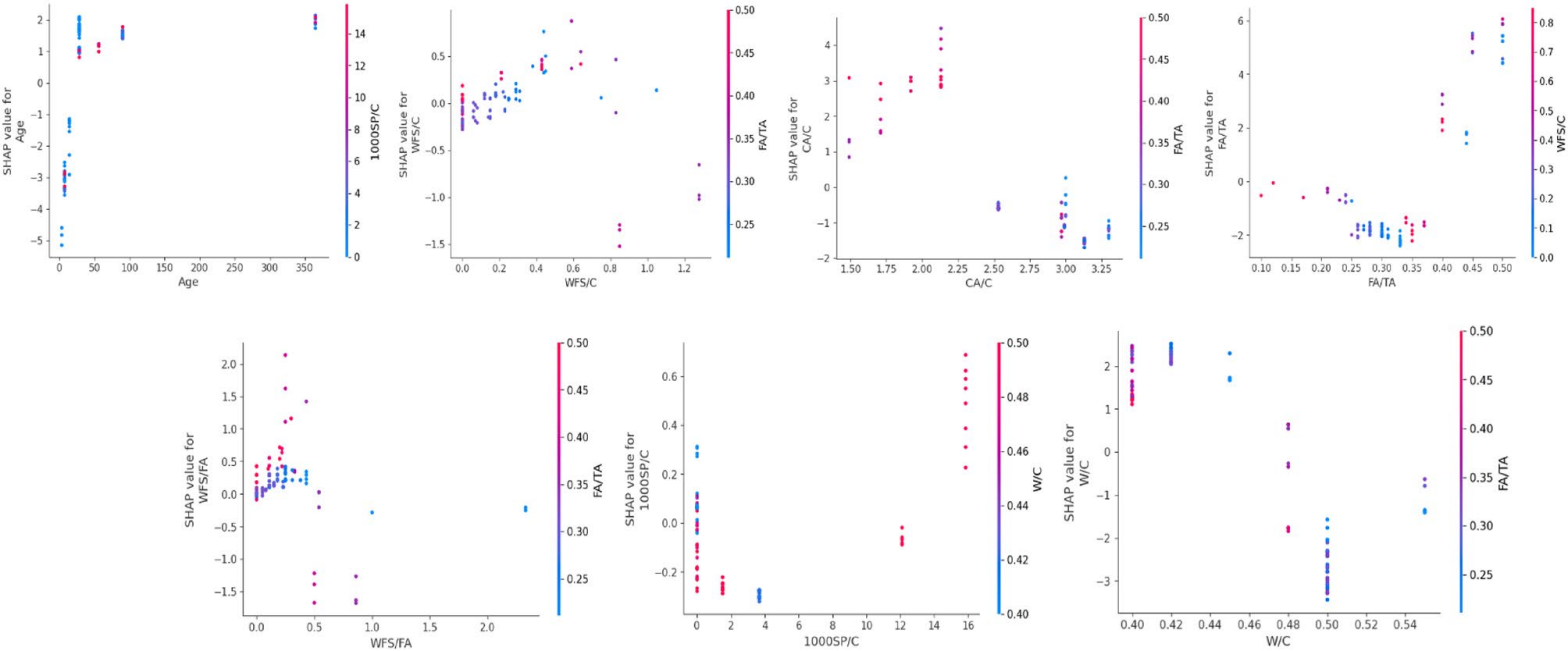
Features interaction plots for elastic modulus model.

**Figure 20:**
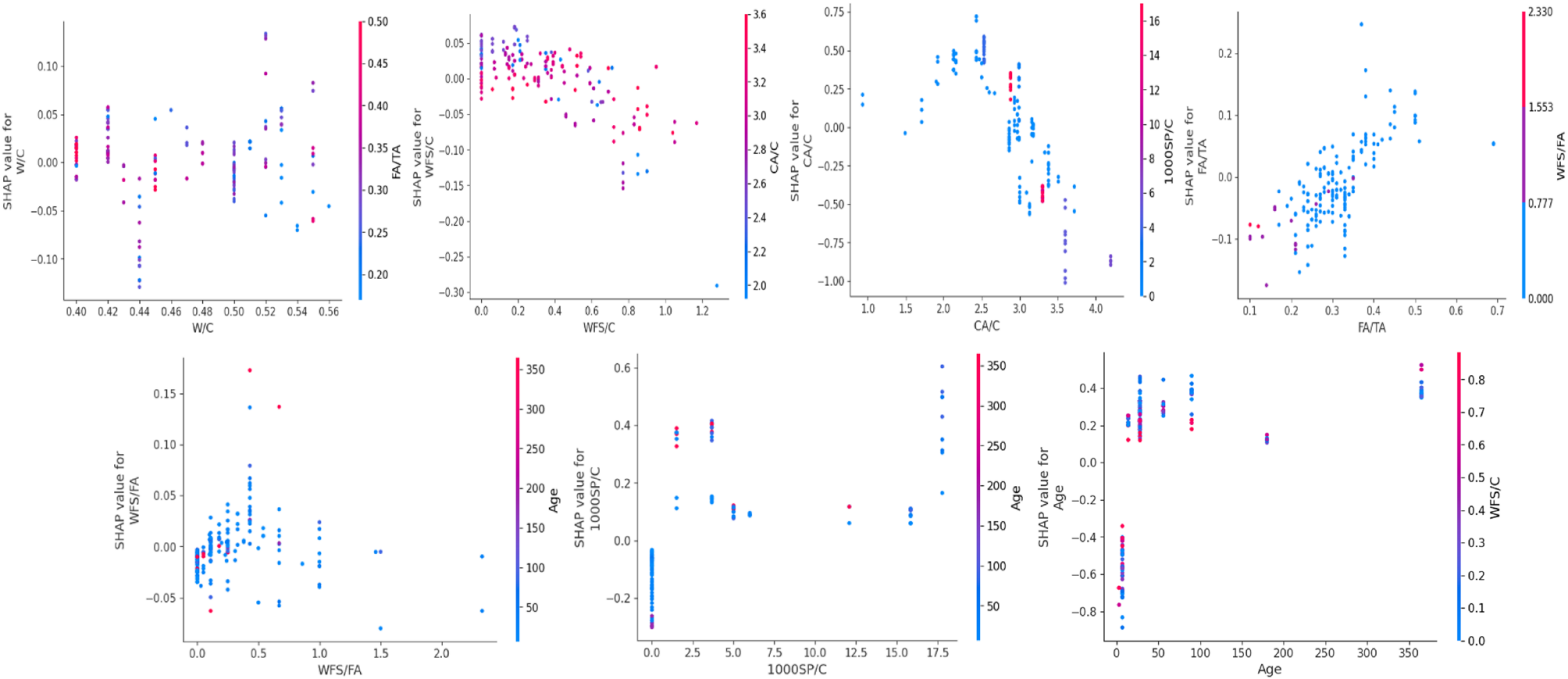
Features interaction plots for split tensile strength model.

While the global SHAP perspective explains the relative significance of contributing factors and their influence on the target variable, it lacks specifics on how each variable impacts the target variable as its value changes. Local interpretation using SHAP analysis is required to optimize the values of input parameters. SHAP local analysis provides a more in-depth knowledge of variable contributions, allowing for the determination of optimal input values for maximizing the target variable. Accordingly, the local explanation is provided in Figs. 18, 19 and 20.

The features interaction plots for compressive strength are provided in Fig. 18. The inclusion of WFS/C positively influences the compressive strength (CS) at a ratio of 0.4. However, increasing the ratio beyond this point leads to a decrease in compressive strength. Figure 18 illustrates that a water-cement ratio of up to 0.5 exhibits a beneficial impact on CS. Moreover, the optimum ratio of CA/C for higher compressive strength is 0.25. The ratio optimum of fine aggregate to coarse aggregate ranges from 0.2 to 0.35. It can be noticed that the optimum ratio of waste foundry sand concrete to fine aggregate is up to 0.4. Siddique et al.^93^ WFS into concrete as a sand replacement, up to 30%, consistently increased in strength. This improvement was attributed to two factors: the densification of the concrete matrix due to the presence of finer WFS particles and the silica content of WFS, which facilitated the formation of C–S–H gel^22^. Similarly, Pathariya et al.^119^ summarized similar findings, observing a consistent increase in strength even at higher replacement levels, specifically up to 60% of WFS. The study reported that the highest strength was achieved in the concrete mixture with 60% WFS. In another study, Singh et al.^23^ reported a maximum compressive strength at 15% replacement of FA with WFS. However, the SHAP analysis provided 25% of fine aggregate replacement with waste foundry sand. Moreover, the 1000SP/C value up to 5 demonstrated enhancement in CS; however, further addition showed no prominent trend. The age interaction plot demonstrates a positive correlation between the compressive strength of the concrete and its age. This is evident from the observed increase in SHAP values at the 365-day mark, indicating a higher contribution of age to the overall compressive strength. These results align with established knowledge in the field, where concrete gains strength over time due to ongoing hydration and the formation of robust cementitious bonds.

Similarly, the features interaction plots for elastic modulus are illustrated in Fig. 19. The elastic modulus of WFSC exhibits an upward trend with increasing age, as indicated by consistently higher positive SHAP values across various age values. This observation implies that the elastic modulus of WFSC gradually improves over time. For instance, Siddique et al.^93^ reported that the modulus of elasticity of the concrete mixes demonstrated a progressive increase over time, with the extent of the increase ranging from 5.2 to 12%, depending on the age of testing and WFS replacement. The optimum ratio of WFS to cement is 0.2 for maximum elastic modulus; however, a higher ratio reduces compressive strength, as shown in Fig. 19. Similarly, the optimum levels for CA/C and FA/TA are 2.25 and 0.3, respectively. Furthermore, WFS/FA content provides a higher SHAP value at approximately 0.35, indicating that the fine aggregate replacement with 25% of waste foundry sand improves elastic modulus. The literature reported an improvement in the elastic modulus when 35% of the fine aggregate is replaced with waste foundry sand^24,88,96^. The optimum water-to-cement ratio for gaining maximum elastic modulus is 0.48.

Furthermore, the features interaction plots for split tensile strength are given in Fig. 20. It can be observed that the optimum WFS/C content level of 0.2 achieved more split tensile strength. Moreover, the STS improves until the content level 3 for CA/C. The ratio of waste foundry sand to fine aggregate is 0.2 for achieving more enhanced split tensile strength. It indicated that replacing fine aggregate with 15% of waste foundry sand enhances the STS; however, a further increase may result in reduced split tensile strength of WFSC. Similar observations were found in experimental studies. For instance, Siddique et al.^23^ examined the incorporation of WFS as a partial replacement for fine aggregate in concrete. They conducted experiments using replacement ratios of 10%, 20%, and 30% and observed a consistent improvement in split tensile strength compared to the control concrete. The enhancements were found to be up to 12%, 14%, and 20%, respectively, corresponding to the respective replacement ratios. Furthermore, Guney et al.^20^ stated a strength increase when WFS was incorporated as a replacement in concrete. The study revealed that strength improvement was observed up to a 10% WFS replacement ratio. However, further substitution at a 15% level resulted in a subsequent decrease in split tensile strength.

**Figure 21:**
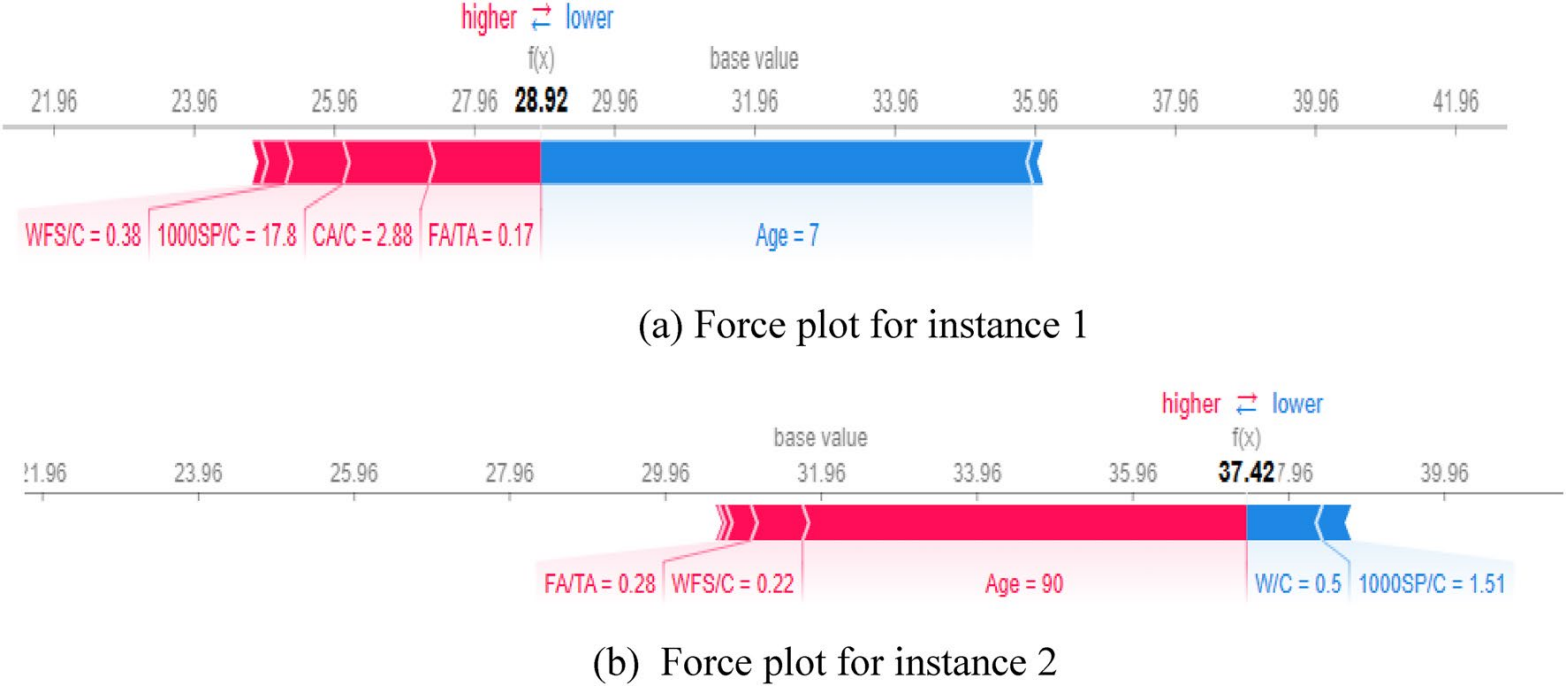
Force plots for compressive strength: (**a**) Instance 1, (**b**) Instance 2.

**Figure 22:**
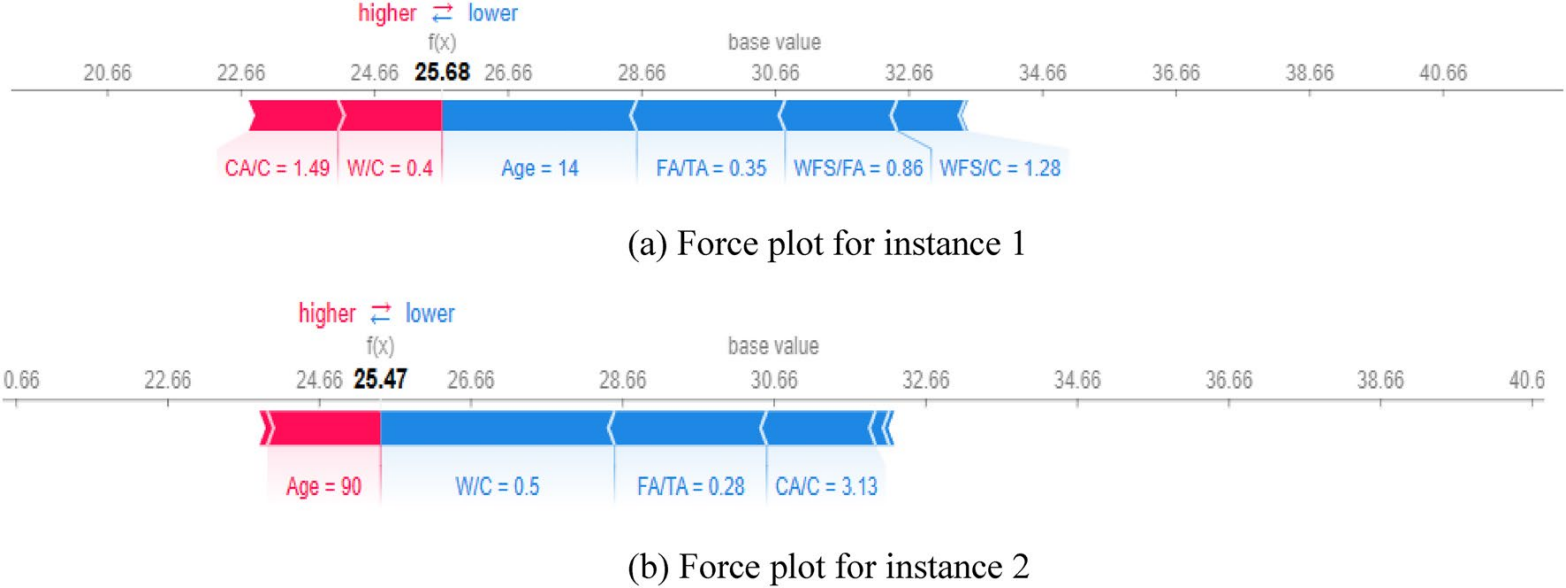
Force plots for elastic modulus: (**a**) Instance 1, (**b**) Instance 2.

**Figure 23:**
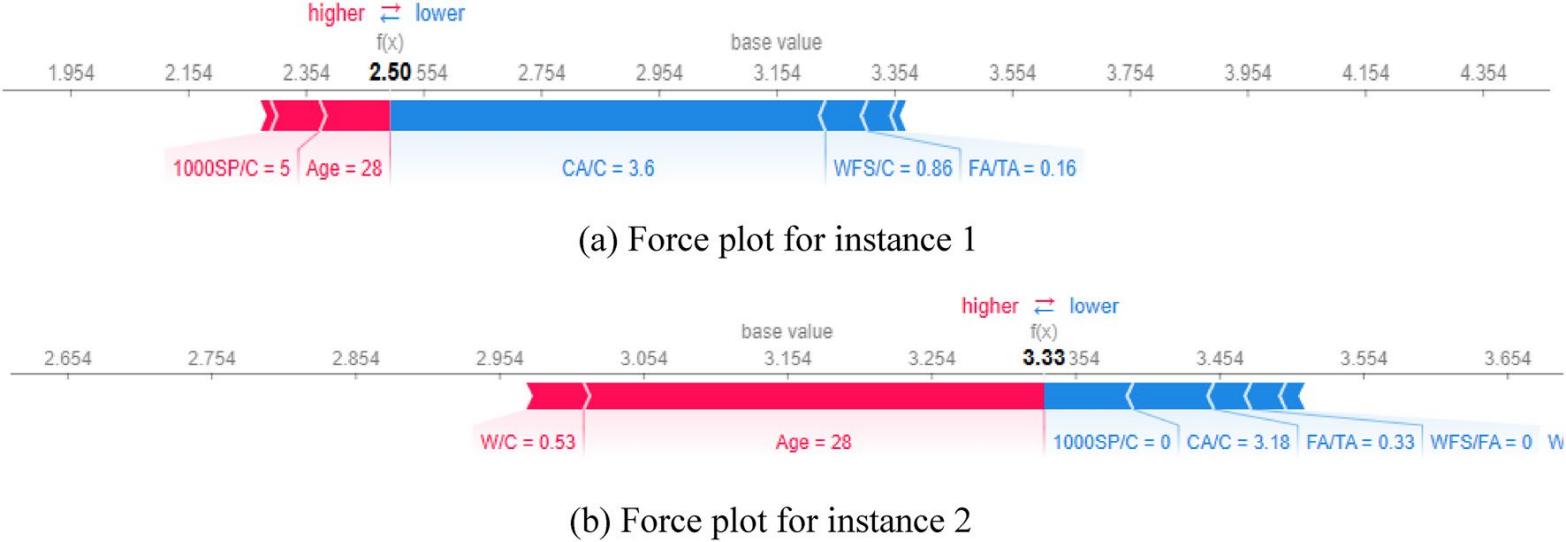
Force plots for split tensile strength: (**a**) Instance 1, (**b**) Instance 2.

Figures 21, 22, 23 provide local explanations for specific predictions through the SHAP force plot and the features interaction interpretation. The plot illustrates two selected instances to offer insights into the individual predictions. This visualization allows for a more focused analysis of the impact of different features on specific predictions, providing a detailed understanding of the factors driving those particular outcomes. In these plots, the bolded values show the output prediction obtained at a particular moment during the model’s training process.

The instance 1 force plot compressive strength illustrates that at the age of 7 days, the compressive strength of WFSC is very low, as indicated by the large blue width of the plot. In contrast, the WFSC gains more compressive strength at 90 days, as shown in Fig. 26b. Moreover, a WFC/C value of 0.38 (Fig. 21a) has a lower positive impact on CS than the content level of 0.22 (Fig. 21b). Similarly, the force plots of elastic modulus are provided in Fig. 22. Age similarly impacts elastic modulus, as indicated by age influence on the E value at 14 days (Fig. 22a) and 90 days (Fig. 22b). In addition, it can be noticed that a content level of 0.86 for WFS/Fa negatively influences the elastic modulus. Furthermore, the positive influence of the superplasticizer can be observed on split tensile strength when the content level of the superplasticizer increases from 0 to 5, as shown in Fig. 23.

In conclusion, the SHAP interpretation is in closer agreement with the outcomes of experimental studies, demonstrating its effectiveness in providing insights into the inner workings of machine learning algorithms. By employing post-hoc explanatory techniques like SHAP, the black-box nature of these models can be unraveled, facilitating a deeper understanding of their functioning even for non-technical individuals. This bridging the gap between technical and non-technical personnel holds promise for promoting transparency, trust, and wider adoption of machine learning models in civil engineering.

### Limitations of the study and recommendation for future research

In the current study, a dataset comprising 397 records was employed to make forecasts for compressive strength, 146 records were utilized for predicting elastic modulus, and 242 records were used for split tensile strength predictions. To enhance model accuracy, future research could emphasize the integration of extra data from the literature. Expanding the database in this manner can improve predictive performance, thereby bolstering the models' robustness and reliability. Moreover, the developed models are only applicable to the considered variables and curing conditions, and deviation from these may necessitate further calibration or validation to ensure the models' reliability and accuracy. Future research might employ other hybrid ML approaches such as random forest with artificial neural networks (RF-ANN) and SVR with particle swarm optimization (SVR-PSO). The adoption of these hybrid approaches holds promise for further refining model precision and predictive capabilities. Furthermore, while the study employed the SHAP for model interpretability, alternate interpretability methods like local interpretable model-agnostic explanations (LIME) and partial dependence plots (PDP) could be applied to elucidate model predictions. Furthermore, it is highly recommended to investigate the ML methods prediction for durability assessment of waste foundry sand concrete.

## Conclusion

The study presents the development of two standalone models, namely, support vector regression (SVR) and decision tree (DT) and an ensemble learning model (AR). Moreover, SVR was employed in conjunction with three robust optimization algorithms: the firefly algorithm (FFA), particle swarm optimization (PSO), and grey wolf optimization (GWO), to construct hybrid models. To develop these models, a comprehensive dataset consisting of 397 records for compressive strength (CS), 146 records for elastic modulus (E), and 242 records for split tensile strength (STS) was collected from experimental studies. The performance of the models was rigorously evaluated using diverse statistical metrics, and the interpretability of the model predictions was accomplished by implementing the SHAP technique. The major findings of the study are provided herein:

- All the models developed in this study demonstrated commendable prediction accuracy in estimating the strength properties of WFSC. Notably, the ensemble and hybrid models showcased superior performance, surpassing the predictive accuracy of individual machine-learning models. This outcome underscores the effectiveness of ensemble and hybrid models to achieve excellent predictive capabilities, offering promising prospects for more accurate and reliable predictions for WFSC strength properties.
- The SVR-GWO hybrid model demonstrated exceptional accuracy in predicting waste foundry sand concrete (WFSC) strength characteristics. The SVR-GWO hybrid model exhibited R-values of 0.999 for CS and E, and 0.998 for STS.
- SHAP analysis revealed that age significantly influences estimating the strength properties of WFSC.
- The SHAP interpretation of the data revealed that the maximum replacement of fine aggregate with waste foundry sand for achieving optimal results is about 25% for compressive strength and elastic modulus, and 15% for split tensile strength. These findings suggest that exceeding these respective replacement percentages may lead to a decline in the desired properties of concrete. It is essential to consider these thresholds when determining the appropriate content level of WFS to ensure the desired strength characteristics in concrete structures.
- The application of these sophisticated soft computing prediction techniques holds the potential to stimulate the widespread adoption of WFS in sustainable concrete production, thereby fostering waste reduction and bolstering the adoption of environmentally conscious construction practices.

### Supplementary Information

Supplementary Table S1.

## Data Availability

Data is provided in supplementary information files.
