# Supplementary material for: Comparative analysis of various machine learning algorithms to predict strength properties of sustainable green concrete containing waste foundry sand
Source: Sci Rep. 2024 Jun 25;14:14617. doi: 10.1038/s41598-024-65255-2 (PMC11199582; doi:10.1038/s41598-024-65255-2)
Supplement: Supplementary file 1 — Supplementary Table S1. [file 41598_2024_65255_MOESM1_ESM.docx]

Table S1: Dataset used in the current study for compressive strength

| WFS/C | W/C | CA/C | FA/TA | WFS/FA | 1000SP/C | Age | CS |
| --- | --- | --- | --- | --- | --- | --- | --- |
| 0.35 | 0.5 | 3.6 | 0.26 | 0.25 | 5 | 28 | 32.67402 |
| 0.52 | 0.5 | 3.6 | 0.23 | 0.43 | 5 | 28 | 37.89344 |
| 0.69 | 0.51 | 3.6 | 0.19 | 0.67 | 5 | 28 | 36.48485 |
| 0.86 | 0.53 | 3.6 | 0.16 | 1 | 5 | 28 | 37.07674 |
| 0 | 0.5 | 3.6 | 0.32 | 0 | 5 | 90 | 43.75166 |
| 0.17 | 0.5 | 3.6 | 0.29 | 0.11 | 5 | 90 | 37.48007 |
| 0.35 | 0.5 | 3.6 | 0.26 | 0.25 | 5 | 90 | 40.04203 |
| 0.69 | 0.51 | 3.6 | 0.19 | 0.67 | 5 | 90 | 44.47576 |
| 0.86 | 0.53 | 3.6 | 0.16 | 1 | 5 | 90 | 45.10788 |
| 1.04 | 0.56 | 3.6 | 0.13 | 1.5 | 5 | 90 | 33.22987 |
| 0 | 0.5 | 3.6 | 0.32 | 0 | 5 | 365 | 44.15628 |
| 0.17 | 0.5 | 3.6 | 0.29 | 0.11 | 5 | 365 | 43.25463 |
| 0.52 | 0.5 | 3.6 | 0.23 | 0.43 | 5 | 365 | 53.81399 |
| 0.69 | 0.51 | 3.6 | 0.19 | 0.67 | 5 | 365 | 50.29778 |
| 0.86 | 0.53 | 3.6 | 0.16 | 1 | 5 | 365 | 51.51277 |
| 1.04 | 0.56 | 3.6 | 0.13 | 1.5 | 5 | 365 | 36.19888 |
| 0 | 0.46 | 2.88 | 0.24 | 0 | 17.8 | 7 | 40.86352 |
| 0.25 | 0.52 | 2.88 | 0.2 | 0.25 | 17.8 | 7 | 34.22501 |
| 0.38 | 0.53 | 2.88 | 0.17 | 0.43 | 17.8 | 7 | 31.37189 |
| 0.51 | 0.54 | 2.88 | 0.1 | 1 | 17.8 | 7 | 29.31201 |
| 0.25 | 0.52 | 2.88 | 0.2 | 0.25 | 17.8 | 28 | 39.33128 |
| 0.38 | 0.53 | 2.88 | 0.17 | 0.43 | 17.8 | 28 | 35.92482 |
| 0 | 0.46 | 2.88 | 0.24 | 0 | 17.8 | 56 | 45.11677 |
| 0.25 | 0.52 | 2.88 | 0.2 | 0.25 | 17.8 | 56 | 42.88245 |
| 0.38 | 0.53 | 2.88 | 0.17 | 0.43 | 17.8 | 56 | 39.44136 |
| 0.51 | 0.54 | 2.88 | 0.1 | 1 | 17.8 | 56 | 35.23592 |
| 0.13 | 0.51 | 2.88 | 0.22 | 0.11 | 17.8 | 90 | 44.46019 |
| 0.25 | 0.52 | 2.88 | 0.2 | 0.25 | 17.8 | 90 | 43.02486 |
| 0.38 | 0.53 | 2.88 | 0.17 | 0.43 | 17.8 | 90 | 40.27641 |
| 0 | 0.5 | 2.99 | 0.33 | 0 | 1.51 | 7 | 19.93163 |
| 0.07 | 0.5 | 2.99 | 0.31 | 0.05 | 1.51 | 7 | 22.50834 |
| 0.14 | 0.5 | 2.99 | 0.3 | 0.11 | 1.51 | 7 | 24.0295 |
| 0.29 | 0.5 | 2.99 | 0.26 | 0.25 | 1.51 | 7 | 25.33658 |
| 0 | 0.5 | 2.99 | 0.33 | 0 | 1.51 | 28 | 29.7918 |
| 0.07 | 0.5 | 2.99 | 0.31 | 0.05 | 1.51 | 28 | 33.83554 |
| 0.14 | 0.5 | 2.99 | 0.3 | 0.11 | 1.51 | 28 | 36.01209 |
| 0.29 | 0.5 | 2.99 | 0.26 | 0.25 | 1.51 | 28 | 36.08175 |
| 0.14 | 0.5 | 2.99 | 0.3 | 0.11 | 1.51 | 90 | 40.98052 |
| 0.22 | 0.5 | 2.99 | 0.28 | 0.18 | 1.51 | 90 | 41.82162 |
| 0 | 0.5 | 2.99 | 0.33 | 0 | 1.51 | 365 | 37.08308 |
| 0.29 | 0.5 | 2.99 | 0.26 | 0.25 | 1.51 | 365 | 44.14715 |
| 0.15 | 0.5 | 3.3 | 0.27 | 0.11 | 15.86 | 7 | 21.81616 |
| 0.29 | 0.5 | 3.3 | 0.24 | 0.25 | 15.86 | 7 | 21.8851 |
| 0.44 | 0.5 | 3.3 | 0.21 | 0.43 | 15.86 | 7 | 22.73998 |
| 0 | 0.5 | 3.3 | 0.3 | 0 | 12.1 | 28 | 26.90399 |
| 0.15 | 0.5 | 3.3 | 0.27 | 0.11 | 15.86 | 28 | 28.43267 |
| 0.29 | 0.5 | 3.3 | 0.24 | 0.25 | 15.86 | 28 | 28.92362 |
| 0.44 | 0.5 | 3.3 | 0.21 | 0.43 | 15.86 | 28 | 29.82237 |
| 0.15 | 0.5 | 3.3 | 0.27 | 0.11 | 15.86 | 56 | 33.33652 |
| 0.29 | 0.5 | 3.3 | 0.24 | 0.25 | 15.86 | 90 | 34.67028 |
| 0.44 | 0.5 | 3.3 | 0.21 | 0.43 | 15.86 | 90 | 36.44699 |
| 0 | 0.5 | 3.3 | 0.3 | 0 | 12.1 | 365 | 33.70306 |
| 0.15 | 0.5 | 3.3 | 0.27 | 0.11 | 15.86 | 365 | 35.19015 |
| 0.29 | 0.5 | 3.3 | 0.24 | 0.25 | 15.86 | 365 | 36.09906 |
| 0.44 | 0.5 | 3.3 | 0.21 | 0.43 | 15.86 | 365 | 37.548 |
| 0.06 | 0.4 | 2.53 | 0.31 | 0.05 | 3.67 | 7 | 29.36908 |
| 0.12 | 0.4 | 2.53 | 0.3 | 0.11 | 3.67 | 7 | 30.76195 |
| 0.18 | 0.4 | 2.53 | 0.28 | 0.18 | 3.67 | 7 | 31.78415 |
| 0.25 | 0.4 | 2.53 | 0.26 | 0.25 | 3.67 | 7 | 31.726 |
| 0 | 0.4 | 2.53 | 0.33 | 0 | 3.67 | 28 | 39.13723 |
| 0.12 | 0.4 | 2.53 | 0.3 | 0.11 | 3.67 | 28 | 44.5601 |
| 0.18 | 0.4 | 2.53 | 0.28 | 0.18 | 3.67 | 28 | 45.63657 |
| 0.25 | 0.4 | 2.53 | 0.26 | 0.25 | 3.67 | 28 | 44.57759 |
| 0 | 0.4 | 2.53 | 0.33 | 0 | 3.67 | 90 | 42.96437 |
| 0.06 | 0.4 | 2.53 | 0.31 | 0.05 | 3.67 | 90 | 45.79331 |
| 0.25 | 0.4 | 2.53 | 0.26 | 0.25 | 3.67 | 90 | 46.80877 |
| 0.31 | 0.5 | 3.13 | 0.26 | 0.31 | 0 | 7 | 18.24256 |
| 0.38 | 0.5 | 3.13 | 0.25 | 0.38 | 0 | 7 | 16.26847 |
| 0.08 | 0.5 | 3.13 | 0.31 | 0.08 | 0 | 14 | 19.94237 |
| 0.15 | 0.5 | 3.13 | 0.3 | 0.15 | 0 | 14 | 20.45881 |
| 0.23 | 0.5 | 3.13 | 0.28 | 0.23 | 0 | 14 | 21.46914 |
| 0.31 | 0.5 | 3.13 | 0.26 | 0.31 | 0 | 14 | 21.23738 |
| 0.38 | 0.5 | 3.13 | 0.25 | 0.38 | 0 | 14 | 18.94789 |
| 0 | 0.5 | 3.13 | 0.33 | 0 | 0 | 21 | 21.93646 |
| 0.08 | 0.5 | 3.13 | 0.31 | 0.08 | 0 | 21 | 22.98048 |
| 0.15 | 0.5 | 3.13 | 0.3 | 0.15 | 0 | 21 | 23.51098 |
| 0.23 | 0.5 | 3.13 | 0.28 | 0.23 | 0 | 21 | 24.40312 |
| 0.31 | 0.5 | 3.13 | 0.26 | 0.31 | 0 | 21 | 23.94155 |
| 0 | 0.5 | 3.13 | 0.33 | 0 | 0 | 28 | 24.34602 |
| 0.08 | 0.5 | 3.13 | 0.31 | 0.08 | 0 | 28 | 25.67314 |
| 0.15 | 0.5 | 3.13 | 0.3 | 0.15 | 0 | 28 | 26.21941 |
| 0.23 | 0.5 | 3.13 | 0.28 | 0.23 | 0 | 28 | 26.98834 |
| 0.31 | 0.5 | 3.13 | 0.26 | 0.31 | 0 | 28 | 26.32684 |
| 0.38 | 0.5 | 3.13 | 0.25 | 0.38 | 0 | 28 | 23.80612 |
| 0 | 0.5 | 4 | 0.33 | 0 | 0 | 1 | 19.2541 |
| 0.6 | 0.5 | 4 | 0.23 | 0.3 | 0 | 1 | 15.95907 |
| 1.2 | 0.5 | 4 | 0.13 | 1.5 | 0 | 1 | 11.43589 |
| 0 | 0.5 | 4 | 0.33 | 0 | 0 | 7 | 24.55064 |
| 0.6 | 0.5 | 4 | 0.23 | 0.3 | 0 | 7 | 19.87051 |
| 1.2 | 0.5 | 4 | 0.13 | 1.5 | 0 | 7 | 15.29043 |
| 0 | 0.5 | 4 | 0.33 | 0 | 0 | 28 | 40.51761 |
| 0.6 | 0.5 | 4 | 0.23 | 0.3 | 0 | 28 | 31.35581 |
| 0.6 | 0.5 | 4 | 0.23 | 0.3 | 0 | 90 | 36.37609 |
| 1.2 | 0.5 | 4 | 0.13 | 1.5 | 0 | 90 | 27.47157 |
| 0 | 0.44 | 2.86 | 0.35 | 0 | 0 | 7 | 22.24645 |
| 0.77 | 0.44 | 2.86 | 0.21 | 1 | 0 | 7 | 16.75725 |
| 0 | 0.44 | 2.86 | 0.35 | 0 | 0 | 28 | 31.73537 |
| 0.31 | 0.44 | 2.86 | 0.3 | 0.25 | 0 | 28 | 32.11696 |
| 0.77 | 0.44 | 2.86 | 0.21 | 1 | 0 | 28 | 25.12064 |
| 0 | 0.44 | 2.86 | 0.35 | 0 | 0 | 90 | 37.41908 |
| 0.15 | 0.44 | 2.86 | 0.33 | 0.11 | 0 | 90 | 37.1824 |
| 0.31 | 0.44 | 2.86 | 0.3 | 0.25 | 0 | 90 | 36.26275 |
| 0.46 | 0.44 | 2.86 | 0.27 | 0.43 | 0 | 90 | 34.35706 |
| 0.77 | 0.44 | 2.86 | 0.21 | 1 | 0 | 90 | 28.10468 |
| 0.15 | 0.44 | 2.86 | 0.33 | 0.11 | 0 | 180 | 36.55549 |
| 0.31 | 0.44 | 2.86 | 0.3 | 0.25 | 0 | 180 | 36.03488 |
| 0.46 | 0.44 | 2.86 | 0.27 | 0.43 | 0 | 180 | 34.48264 |
| 0.61 | 0.44 | 2.86 | 0.24 | 0.67 | 0 | 180 | 31.36182 |
| 0.77 | 0.44 | 2.86 | 0.21 | 1 | 0 | 180 | 27.93289 |
| 0 | 0.47 | 2.47 | 0.38 | 0 | 0 | 28 | 33.27887 |
| 0.46 | 0.47 | 2.47 | 0.3 | 0.43 | 0 | 28 | 37.42341 |
| 0.61 | 0.47 | 2.47 | 0.27 | 0.67 | 0 | 28 | 39.08365 |
| 0.77 | 0.47 | 2.47 | 0.24 | 1 | 0 | 28 | 36.39465 |
| 0.68 | 0.47 | 2.75 | 0.26 | 0.67 | 0 | 28 | 38.37459 |
| 0.77 | 0.47 | 3.09 | 0.26 | 0.67 | 0 | 28 | 31.13757 |
| 0.68 | 0.47 | 2.75 | 0.26 | 0.67 | 0 | 28 | 38.37459 |
| 0 | 0.52 | 4.2 | 0.38 | 0 | 6 | 7 | 15.42529 |
| 0.25 | 0.52 | 4.2 | 0.34 | 0.11 | 6 | 7 | 15.6392 |
| 0.51 | 0.52 | 4.2 | 0.3 | 0.25 | 6 | 7 | 23.17605 |
| 0.76 | 0.52 | 4.2 | 0.26 | 0.43 | 6 | 7 | 19.06639 |
| 0.03 | 0.52 | 4.2 | 0.35 | 0.01 | 6 | 7 | 19.47432 |
| 0.06 | 0.52 | 4.2 | 0.32 | 0.03 | 6 | 7 | 19.05294 |
| 0.09 | 0.52 | 4.2 | 0.29 | 0.05 | 6 | 7 | 15.30296 |
| 0 | 0.52 | 4.2 | 0.38 | 0 | 6 | 28 | 29.01128 |
| 0.25 | 0.52 | 4.2 | 0.34 | 0.11 | 6 | 28 | 21.9171 |
| 0.76 | 0.52 | 4.2 | 0.26 | 0.43 | 6 | 28 | 29.33813 |
| 0.03 | 0.52 | 4.2 | 0.35 | 0.01 | 6 | 28 | 30.49103 |
| 0.09 | 0.52 | 4.2 | 0.29 | 0.05 | 6 | 28 | 22.49364 |
| 0 | 0.47 | 2.98 | 0.33 | 0 | 0 | 28 | 32.28385 |
| 0.29 | 0.47 | 2.98 | 0.26 | 0.25 | 0 | 28 | 29.44638 |
| 0.29 | 0.47 | 2.98 | 0.3 | 0.11 | 0 | 28 | 34.73054 |
| 0.15 | 0.47 | 2.98 | 0.28 | 0.18 | 0 | 28 | 32.15924 |
| 0.31 | 0.44 | 2.86 | 0.3 | 0.25 | 0 | 7 | 22.88726 |
| 0.46 | 0.44 | 2.86 | 0.27 | 0.43 | 0 | 7 | 20.54631 |
| 0.77 | 0.44 | 2.86 | 0.21 | 1 | 0 | 7 | 16.75725 |
| 0 | 0.44 | 2.86 | 0.35 | 0 | 0 | 28 | 31.73537 |
| 0.31 | 0.44 | 2.86 | 0.3 | 0.25 | 0 | 28 | 32.11696 |
| 0.46 | 0.44 | 2.86 | 0.27 | 0.43 | 0 | 28 | 30.69222 |
| 0.61 | 0.44 | 2.86 | 0.24 | 0.67 | 0 | 28 | 29.2833 |
| 0.77 | 0.44 | 2.86 | 0.21 | 1 | 0 | 28 | 25.12064 |
| 0 | 0.44 | 2.86 | 0.35 | 0 | 0 | 90 | 37.41908 |
| 0.31 | 0.44 | 2.86 | 0.3 | 0.25 | 0 | 90 | 36.26275 |
| 0.46 | 0.44 | 2.86 | 0.27 | 0.43 | 0 | 90 | 34.35706 |
| 0.61 | 0.44 | 2.86 | 0.24 | 0.67 | 0 | 90 | 32.17759 |
| 0.77 | 0.44 | 2.86 | 0.21 | 1 | 0 | 90 | 28.10468 |
| 0 | 0.44 | 2.86 | 0.35 | 0 | 0 | 180 | 37.17376 |
| 0.15 | 0.44 | 2.86 | 0.33 | 0.11 | 0 | 180 | 36.55549 |
| 0.31 | 0.44 | 2.86 | 0.3 | 0.25 | 0 | 180 | 36.03488 |
| 0.46 | 0.44 | 2.86 | 0.27 | 0.43 | 0 | 180 | 34.48264 |
| 0.61 | 0.44 | 2.86 | 0.24 | 0.67 | 0 | 180 | 31.36182 |
| 0 | 0.4 | 2.5 | 0.29 | 0 | 0 | 7 | 27.83476 |
| 0.2 | 0.4 | 2.5 | 0.24 | 0.25 | 0 | 7 | 27.24942 |
| 0.4 | 0.4 | 2.5 | 0.2 | 0.66 | 0 | 7 | 27.41909 |
| 0.2 | 0.4 | 2.5 | 0.24 | 0.25 | 0 | 14 | 31.40966 |
| 0.4 | 0.4 | 2.5 | 0.2 | 0.66 | 0 | 14 | 31.43574 |
| 0.6 | 0.4 | 2.5 | 0.14 | 1.46 | 0 | 14 | 35.84802 |
| 0 | 0.4 | 2.5 | 0.29 | 0 | 0 | 28 | 36.90842 |
| 0.2 | 0.4 | 2.5 | 0.24 | 0.25 | 0 | 28 | 38.59465 |
| 0.4 | 0.4 | 2.5 | 0.2 | 0.66 | 0 | 28 | 38.5961 |
| 0.6 | 0.4 | 2.5 | 0.14 | 1.46 | 0 | 28 | 40.06072 |
| 0 | 0.5 | 3.38 | 0.35 | 0 | 0 | 7 | 21.74541 |
| 0.18 | 0.5 | 3.38 | 0.33 | 0.11 | 0 | 7 | 23.46413 |
| 0.54 | 0.5 | 3.38 | 0.27 | 0.43 | 0 | 7 | 25.45474 |
| 0.72 | 0.5 | 3.38 | 0.24 | 0.67 | 0 | 7 | 24.47538 |
| 0.18 | 0.5 | 3.38 | 0.33 | 0.11 | 0 | 28 | 29.51399 |
| 0.36 | 0.5 | 3.38 | 0.3 | 0.25 | 0 | 28 | 30.48062 |
| 0.54 | 0.5 | 3.38 | 0.27 | 0.43 | 0 | 28 | 31.23181 |
| 0.72 | 0.5 | 3.38 | 0.24 | 0.67 | 0 | 28 | 30.04937 |
| 0 | 0.5 | 3.38 | 0.35 | 0 | 0 | 56 | 32.17724 |
| 0.18 | 0.5 | 3.38 | 0.33 | 0.11 | 0 | 56 | 34.09604 |
| 0.36 | 0.5 | 3.38 | 0.3 | 0.25 | 0 | 56 | 35.23284 |
| 0.72 | 0.5 | 3.38 | 0.24 | 0.67 | 0 | 56 | 34.86203 |
| 0.36 | 0.5 | 3.38 | 0.3 | 0.25 | 0 | 90 | 37.76259 |
| 0.72 | 0.5 | 3.38 | 0.24 | 0.67 | 0 | 90 | 37.40461 |
| 0.27 | 0.43 | 2.99 | 0.34 | 0.18 | 0 | 7 | 32.6905 |
| 0.36 | 0.43 | 2.99 | 0.32 | 0.25 | 0 | 7 | 29.98844 |
| 0 | 0.43 | 2.8 | 0.34 | 0 | 0 | 7 | 24.08326 |
| 0.22 | 0.43 | 2.8 | 0.31 | 0.18 | 0 | 7 | 31.92665 |
| 0.29 | 0.43 | 2.8 | 0.3 | 0.25 | 0 | 7 | 30.48191 |
| 0.09 | 0.43 | 2.99 | 0.36 | 0.05 | 0 | 28 | 33.94429 |
| 0.18 | 0.43 | 2.99 | 0.35 | 0.11 | 0 | 28 | 37.01209 |
| 0.36 | 0.43 | 2.99 | 0.32 | 0.25 | 0 | 28 | 36.69602 |
| 0 | 0.43 | 2.8 | 0.34 | 0 | 0 | 28 | 31.3041 |
| 0.07 | 0.43 | 2.8 | 0.33 | 0.05 | 0 | 28 | 33.63281 |
| 0.15 | 0.43 | 2.8 | 0.32 | 0.11 | 0 | 28 | 36.46047 |
| 0.22 | 0.43 | 2.8 | 0.31 | 0.18 | 0 | 28 | 38.50233 |
| 0.29 | 0.43 | 2.8 | 0.3 | 0.25 | 0 | 28 | 37.80723 |
| 0 | 0.42 | 3.16 | 0.38 | 0 | 0 | 7 | 27.86811 |
| 0.78 | 0.42 | 3.16 | 0.27 | 0.67 | 0 | 7 | 28.93773 |
| 1.17 | 0.42 | 3.16 | 0.2 | 1.5 | 0 | 7 | 23.96472 |
| 0.95 | 0.42 | 3.33 | 0.38 | 0 | 0 | 7 | 27.21687 |
| 0.9 | 0.42 | 3.51 | 0.38 | 0 | 0 | 7 | 24.5053 |
| 0 | 0.42 | 3.16 | 0.38 | 0 | 0 | 14 | 29.98026 |
| 0.78 | 0.42 | 3.16 | 0.27 | 0.67 | 0 | 14 | 31.55506 |
| 0.95 | 0.42 | 3.33 | 0.38 | 0 | 0 | 14 | 28.98061 |
| 0.85 | 0.42 | 3.72 | 0.38 | 0 | 0 | 14 | 22.68531 |
| 0 | 0.42 | 3.16 | 0.38 | 0 | 0 | 28 | 33.42797 |
| 0.39 | 0.42 | 3.16 | 0.33 | 0.25 | 0 | 28 | 33.19931 |
| 0.78 | 0.42 | 3.16 | 0.27 | 0.67 | 0 | 28 | 35.73014 |
| 1.17 | 0.42 | 3.16 | 0.2 | 1.5 | 0 | 28 | 27.09081 |
| 0.9 | 0.42 | 3.51 | 0.38 | 0 | 0 | 28 | 28.64315 |
| 0 | 0.45 | 0.93 | 0.69 | 0 | 0 | 7 | 21.11722 |
| 0.42 | 0.45 | 0.93 | 0.69 | 0.25 | 0 | 7 | 33.96377 |
| 0.63 | 0.45 | 0.93 | 0.69 | 0.43 | 0 | 7 | 23.03392 |
| 0 | 0.45 | 1.9 | 0.51 | 0 | 0 | 7 | 22.28243 |
| 0.2 | 0.45 | 1.9 | 0.51 | 0.11 | 0 | 7 | 25.34883 |
| 0.4 | 0.45 | 1.9 | 0.51 | 0.25 | 0 | 7 | 23.11952 |
| 0.59 | 0.45 | 1.9 | 0.51 | 0.43 | 0 | 7 | 22.37968 |
| 0 | 0.45 | 0.93 | 0.69 | 0 | 0 | 28 | 26.45342 |
| 0.21 | 0.45 | 0.93 | 0.69 | 0.11 | 0 | 28 | 32.13758 |
| 0.42 | 0.45 | 0.93 | 0.69 | 0.25 | 0 | 28 | 36.68415 |
| 0.63 | 0.45 | 0.93 | 0.69 | 0.43 | 0 | 28 | 27.38554 |
| 0.2 | 0.45 | 1.9 | 0.51 | 0.11 | 0 | 28 | 29.70915 |
| 0.4 | 0.45 | 1.9 | 0.51 | 0.25 | 0 | 28 | 23.5357 |
| 0.59 | 0.45 | 1.9 | 0.51 | 0.43 | 0 | 28 | 21.77642 |
| 0 | 0.53 | 3.18 | 0.33 | 0 | 0 | 28 | 36.23349 |
| 0.08 | 0.53 | 3.18 | 0.32 | 0.05 | 0 | 28 | 38.46498 |
| 0.16 | 0.53 | 3.18 | 0.3 | 0.11 | 0 | 28 | 40.21604 |
| 0.24 | 0.53 | 3.18 | 0.28 | 0.18 | 0 | 28 | 42.41388 |
| 0.32 | 0.53 | 3.18 | 0.27 | 0.25 | 0 | 28 | 44.68221 |
| 0.48 | 0.53 | 3.18 | 0.23 | 0.43 | 0 | 28 | 48.14485 |
| 0.56 | 0.53 | 3.18 | 0.22 | 0.54 | 0 | 28 | 48.55534 |
| 0.64 | 0.53 | 3.18 | 0.2 | 0.67 | 0 | 28 | 48.05464 |
| 0.12 | 0.43 | 2.7 | 0.28 | 0.11 | 0 | 28 | 31.16772 |
| 0.62 | 0.43 | 2.7 | 0.16 | 1 | 0 | 28 | 29.39399 |
| 0 | 0.52 | 2.93 | 0.37 | 0 | 0 | 7 | 22.69816 |
| 0.17 | 0.52 | 2.93 | 0.33 | 0.11 | 0 | 7 | 23.96578 |
| 0.35 | 0.52 | 2.93 | 0.3 | 0.25 | 0 | 7 | 25.01932 |
| 0 | 0.52 | 2.93 | 0.37 | 0 | 0 | 28 | 30.8864 |
| 0.17 | 0.52 | 2.93 | 0.33 | 0.11 | 0 | 28 | 33.74211 |
| 0.35 | 0.52 | 2.93 | 0.3 | 0.25 | 0 | 28 | 34.17169 |
| 0.52 | 0.52 | 2.93 | 0.26 | 0.43 | 0 | 28 | 32.66409 |
| 0.69 | 0.52 | 2.93 | 0.22 | 0.67 | 0 | 28 | 32.17398 |
| 0 | 0.55 | 3 | 0.33 | 0 | 0 | 7 | 26.75943 |
| 0.15 | 0.55 | 3 | 0.3 | 0.11 | 0 | 7 | 28.11613 |
| 0.45 | 0.55 | 3 | 0.23 | 0.43 | 0 | 7 | 30.70416 |
| 0.75 | 0.55 | 3 | 0.17 | 1 | 0 | 7 | 25.99148 |
| 0 | 0.45 | 3 | 0.25 | 0 | 0 | 7 | 34.57522 |
| 0.15 | 0.45 | 3 | 0.24 | 0.11 | 0 | 7 | 36.94022 |
| 0 | 0.55 | 3 | 0.33 | 0 | 0 | 28 | 34.90407 |
| 0.15 | 0.55 | 3 | 0.3 | 0.11 | 0 | 28 | 36.15811 |
| 0.45 | 0.55 | 3 | 0.23 | 0.43 | 0 | 28 | 37.93065 |
| 0.75 | 0.55 | 3 | 0.17 | 1 | 0 | 28 | 33.11362 |
| 1.05 | 0.55 | 3 | 0.1 | 2.33 | 0 | 28 | 35.70399 |
| 0 | 0.45 | 3 | 0.25 | 0 | 0 | 28 | 45.4417 |
| 0.15 | 0.45 | 3 | 0.24 | 0.11 | 0 | 28 | 48.80081 |
| 0.75 | 0.45 | 3 | 0.17 | 1 | 0 | 28 | 46.06537 |
| 1.05 | 0.45 | 3 | 0.12 | 2.33 | 0 | 28 | 44.93792 |
| 0 | 0.5 | 2.99 | 0.33 | 0 | 1.51 | 7 | 19.93163 |
| 0.12 | 0.42 | 2.53 | 0.3 | 0.11 | 3.67 | 7 | 30.53931 |
| 0.18 | 0.42 | 2.53 | 0.28 | 0.18 | 3.67 | 7 | 31.65494 |
| 0.25 | 0.42 | 2.53 | 0.26 | 0.25 | 3.67 | 7 | 31.27599 |
| 0 | 0.5 | 2.99 | 0.33 | 0 | 1.51 | 28 | 29.7918 |
| 0.07 | 0.5 | 2.99 | 0.31 | 0.05 | 1.51 | 28 | 33.83554 |
| 0.14 | 0.5 | 2.99 | 0.3 | 0.11 | 1.51 | 28 | 36.01209 |
| 0.22 | 0.5 | 2.99 | 0.28 | 0.18 | 1.51 | 28 | 37.28878 |
| 0.29 | 0.5 | 2.99 | 0.26 | 0.25 | 1.51 | 28 | 36.08175 |
| 0.18 | 0.42 | 2.53 | 0.28 | 0.18 | 3.67 | 28 | 45.76975 |
| 0 | 0.5 | 2.99 | 0.33 | 0 | 1.51 | 90 | 34.8752 |
| 0.14 | 0.5 | 2.99 | 0.3 | 0.11 | 1.51 | 90 | 40.98052 |
| 0.06 | 0.42 | 2.53 | 0.31 | 0.05 | 3.67 | 90 | 45.6839 |
| 0.12 | 0.42 | 2.53 | 0.3 | 0.11 | 3.67 | 90 | 47.30846 |
| 0.25 | 0.42 | 2.53 | 0.26 | 0.25 | 3.67 | 90 | 46.78835 |
| 0 | 0.5 | 2.99 | 0.33 | 0 | 1.51 | 365 | 37.08308 |
| 0.07 | 0.5 | 2.99 | 0.31 | 0.05 | 1.51 | 365 | 40.55843 |
| 0.14 | 0.5 | 2.99 | 0.3 | 0.11 | 1.51 | 365 | 43.48254 |
| 0.22 | 0.5 | 2.99 | 0.28 | 0.18 | 1.51 | 365 | 44.83873 |
| 0.29 | 0.5 | 2.99 | 0.26 | 0.25 | 1.51 | 365 | 44.14715 |
| 0 | 0.42 | 2.53 | 0.33 | 0 | 3.67 | 365 | 45.96671 |
| 0.06 | 0.42 | 2.53 | 0.31 | 0.05 | 3.67 | 365 | 49.6555 |
| 0.18 | 0.42 | 2.53 | 0.28 | 0.18 | 3.67 | 365 | 53.73507 |
| 0 | 0.45 | 2.43 | 0.44 | 0 | 0 | 7 | 30.97614 |
| 0.38 | 0.45 | 2.43 | 0.35 | 0.25 | 0 | 7 | 32.73534 |
| 0.58 | 0.45 | 2.43 | 0.31 | 0.43 | 0 | 7 | 29.053 |
| 0.77 | 0.45 | 2.43 | 0.26 | 1 | 0 | 7 | 26.03631 |
| 0.19 | 0.45 | 2.43 | 0.4 | 0.11 | 0 | 28 | 49.35783 |
| 0.38 | 0.45 | 2.43 | 0.35 | 0.25 | 0 | 28 | 50.46187 |
| 0.77 | 0.45 | 2.43 | 0.26 | 1 | 0 | 28 | 39.77336 |
| 0 | 0.5 | 3.6 | 0.32 | 0 | 5 | 28 | 36.12261 |
| 0.17 | 0.5 | 3.6 | 0.29 | 0.11 | 5 | 28 | 31.13882 |
| 1.04 | 0.56 | 3.6 | 0.13 | 1.5 | 5 | 28 | 29.77051 |
| 0.52 | 0.5 | 3.6 | 0.23 | 0.43 | 5 | 90 | 46.41214 |
| 0.35 | 0.5 | 3.6 | 0.26 | 0.25 | 5 | 365 | 47.16811 |
| 0.13 | 0.51 | 2.88 | 0.22 | 0.11 | 17.8 | 7 | 36.77242 |
| 0 | 0.46 | 2.88 | 0.24 | 0 | 17.8 | 28 | 43.35436 |
| 0.13 | 0.51 | 2.88 | 0.22 | 0.11 | 17.8 | 28 | 41.32208 |
| 0.51 | 0.54 | 2.88 | 0.1 | 1 | 17.8 | 28 | 31.93651 |
| 0.13 | 0.51 | 2.88 | 0.22 | 0.11 | 17.8 | 56 | 44.44616 |
| 0 | 0.46 | 2.88 | 0.24 | 0 | 17.8 | 90 | 45.30923 |
| 0.51 | 0.54 | 2.88 | 0.1 | 1 | 17.8 | 90 | 37.27128 |
| 0.22 | 0.5 | 2.99 | 0.28 | 0.18 | 1.51 | 7 | 25.59032 |
| 0.22 | 0.5 | 2.99 | 0.28 | 0.18 | 1.51 | 28 | 37.28878 |
| 0 | 0.5 | 2.99 | 0.33 | 0 | 1.51 | 90 | 34.8752 |
| 0.07 | 0.5 | 2.99 | 0.31 | 0.05 | 1.51 | 90 | 38.58288 |
| 0.29 | 0.5 | 2.99 | 0.26 | 0.25 | 1.51 | 90 | 41.09792 |
| 0.07 | 0.5 | 2.99 | 0.31 | 0.05 | 1.51 | 365 | 40.55843 |
| 0.14 | 0.5 | 2.99 | 0.3 | 0.11 | 1.51 | 365 | 43.48254 |
| 0.22 | 0.5 | 2.99 | 0.28 | 0.18 | 1.51 | 365 | 44.83873 |
| 0 | 0.5 | 3.3 | 0.3 | 0 | 12.1 | 7 | 20.59708 |
| 0 | 0.5 | 3.3 | 0.3 | 0 | 12.1 | 56 | 31.44109 |
| 0.29 | 0.5 | 3.3 | 0.24 | 0.25 | 15.86 | 56 | 34.07373 |
| 0.44 | 0.5 | 3.3 | 0.21 | 0.43 | 15.86 | 56 | 35.36474 |
| 0 | 0.5 | 3.3 | 0.3 | 0 | 12.1 | 90 | 32.23721 |
| 0.15 | 0.5 | 3.3 | 0.27 | 0.11 | 15.86 | 90 | 33.89888 |
| 0 | 0.4 | 2.53 | 0.33 | 0 | 3.67 | 7 | 27.59425 |
| 0.06 | 0.4 | 2.53 | 0.31 | 0.05 | 3.67 | 28 | 42.53106 |
| 0.12 | 0.4 | 2.53 | 0.3 | 0.11 | 3.67 | 90 | 47.18588 |
| 0.18 | 0.4 | 2.53 | 0.28 | 0.18 | 3.67 | 90 | 47.65664 |
| 0 | 0.5 | 3.13 | 0.33 | 0 | 0 | 7 | 16.19733 |
| 0.08 | 0.5 | 3.13 | 0.31 | 0.08 | 0 | 7 | 16.6068 |
| 0.15 | 0.5 | 3.13 | 0.3 | 0.15 | 0 | 7 | 17.10334 |
| 0.23 | 0.5 | 3.13 | 0.28 | 0.23 | 0 | 7 | 18.22342 |
| 0 | 0.5 | 3.13 | 0.33 | 0 | 0 | 14 | 19.20537 |
| 0.38 | 0.5 | 3.13 | 0.25 | 0.38 | 0 | 21 | 21.46384 |
| 1.2 | 0.5 | 4 | 0.13 | 1.5 | 0 | 28 | 26.66096 |
| 0 | 0.5 | 4 | 0.33 | 0 | 0 | 90 | 48.89272 |
| 0.15 | 0.44 | 2.86 | 0.33 | 0.11 | 0 | 7 | 23.32885 |
| 0.31 | 0.44 | 2.86 | 0.3 | 0.25 | 0 | 7 | 22.88726 |
| 0.46 | 0.44 | 2.86 | 0.27 | 0.43 | 0 | 7 | 20.54631 |
| 0.61 | 0.44 | 2.86 | 0.24 | 0.67 | 0 | 7 | 19.7543 |
| 0.15 | 0.44 | 2.86 | 0.33 | 0.11 | 0 | 28 | 32.62015 |
| 0.46 | 0.44 | 2.86 | 0.27 | 0.43 | 0 | 28 | 30.69222 |
| 0.61 | 0.44 | 2.86 | 0.24 | 0.67 | 0 | 28 | 29.2833 |
| 0.61 | 0.44 | 2.86 | 0.24 | 0.67 | 0 | 90 | 32.17759 |
| 0 | 0.44 | 2.86 | 0.35 | 0 | 0 | 180 | 37.17376 |
| 0.15 | 0.47 | 2.47 | 0.36 | 0.11 | 0 | 28 | 35.12947 |
| 0.31 | 0.47 | 2.47 | 0.33 | 0.25 | 0 | 28 | 36.73584 |
| 0.72 | 0.47 | 2.91 | 0.26 | 0.67 | 0 | 28 | 34.80855 |
| 0.65 | 0.47 | 2.6 | 0.27 | 0.67 | 0 | 28 | 40.36426 |
| 0.66 | 0.47 | 2.67 | 0.27 | 0.67 | 0 | 28 | 39.77948 |
| 0.51 | 0.52 | 4.2 | 0.3 | 0.25 | 6 | 28 | 28.22253 |
| 0.06 | 0.52 | 4.2 | 0.32 | 0.03 | 6 | 28 | 27.25751 |
| 0.15 | 0.47 | 2.98 | 0.3 | 0.11 | 0 | 28 | 33.14731 |
| 0.22 | 0.47 | 2.98 | 0.28 | 0.18 | 0 | 28 | 31.30027 |
| 0.22 | 0.47 | 2.98 | 0.26 | 0.25 | 0 | 28 | 30.95881 |
| 0 | 0.5 | 3.99 | 0.33 | 0 | 0 | 28 | 40.63346 |
| 1.2 | 0.5 | 3.99 | 0.13 | 1.5 | 0 | 28 | 27.01603 |
| 0 | 0.44 | 2.86 | 0.35 | 0 | 0 | 7 | 22.24645 |
| 0.15 | 0.44 | 2.86 | 0.33 | 0.11 | 0 | 7 | 23.32885 |
| 0.61 | 0.44 | 2.86 | 0.24 | 0.67 | 0 | 7 | 19.7543 |
| 0.15 | 0.44 | 2.86 | 0.33 | 0.11 | 0 | 28 | 32.62015 |
| 0.15 | 0.44 | 2.86 | 0.33 | 0.11 | 0 | 90 | 37.1824 |
| 0.77 | 0.44 | 2.86 | 0.21 | 1 | 0 | 180 | 27.93289 |
| 0.6 | 0.4 | 2.5 | 0.14 | 1.46 | 0 | 7 | 33.57376 |
| 0 | 0.4 | 2.5 | 0.29 | 0 | 0 | 14 | 31.22198 |
| 0.36 | 0.5 | 3.38 | 0.3 | 0.25 | 0 | 7 | 24.68758 |
| 0 | 0.5 | 3.38 | 0.35 | 0 | 0 | 28 | 28.00809 |
| 0.54 | 0.5 | 3.38 | 0.27 | 0.43 | 0 | 56 | 36.2649 |
| 0 | 0.5 | 3.38 | 0.35 | 0 | 0 | 90 | 33.47254 |
| 0.18 | 0.5 | 3.38 | 0.33 | 0.11 | 0 | 90 | 36.21111 |
| 0.54 | 0.5 | 3.38 | 0.27 | 0.43 | 0 | 90 | 39.2352 |
| 0 | 0.43 | 2.57 | 0.41 | 0 | 0 | 7 | 22.27964 |
| 0.09 | 0.43 | 2.99 | 0.36 | 0.05 | 0 | 7 | 26.25735 |
| 0.18 | 0.43 | 2.99 | 0.35 | 0.11 | 0 | 7 | 29.98025 |
| 0.07 | 0.43 | 2.8 | 0.33 | 0.05 | 0 | 7 | 26.79923 |
| 0.15 | 0.43 | 2.8 | 0.32 | 0.11 | 0 | 7 | 29.91796 |
| 0 | 0.43 | 2.57 | 0.41 | 0 | 0 | 28 | 32.32232 |
| 0.27 | 0.43 | 2.99 | 0.34 | 0.18 | 0 | 28 | 39.25176 |
| 0.39 | 0.42 | 3.16 | 0.33 | 0.25 | 0 | 7 | 27.69094 |
| 0.85 | 0.42 | 3.72 | 0.38 | 0 | 0 | 7 | 21.82299 |
| 0.39 | 0.42 | 3.16 | 0.33 | 0.25 | 0 | 14 | 29.83416 |
| 1.17 | 0.42 | 3.16 | 0.2 | 1.5 | 0 | 14 | 25.0875 |
| 0.9 | 0.42 | 3.51 | 0.38 | 0 | 0 | 14 | 25.94257 |
| 0.95 | 0.42 | 3.33 | 0.38 | 0 | 0 | 28 | 32.31497 |
| 0.85 | 0.42 | 3.72 | 0.38 | 0 | 0 | 28 | 24.25163 |
| 0.21 | 0.45 | 0.93 | 0.69 | 0.11 | 0 | 7 | 28.16093 |
| 0 | 0.45 | 1.9 | 0.51 | 0 | 0 | 28 | 28.5237 |
| 0.4 | 0.53 | 3.18 | 0.25 | 0.33 | 0 | 28 | 46.79946 |
| 0 | 0.43 | 2.7 | 0.31 | 0 | 0 | 28 | 28.28871 |
| 0.37 | 0.43 | 2.7 | 0.22 | 0.43 | 0 | 28 | 31.52304 |
| 0.52 | 0.52 | 2.93 | 0.26 | 0.43 | 0 | 7 | 24.03959 |
| 0.69 | 0.52 | 2.93 | 0.22 | 0.67 | 0 | 7 | 23.70345 |
| 1.05 | 0.55 | 3 | 0.1 | 2.33 | 0 | 7 | 29.25848 |
| 0.45 | 0.45 | 3 | 0.21 | 0.43 | 0 | 7 | 37.36199 |
| 0.75 | 0.45 | 3 | 0.17 | 1 | 0 | 7 | 37.13295 |
| 1.05 | 0.45 | 3 | 0.12 | 2.33 | 0 | 7 | 36.97307 |
| 0.45 | 0.45 | 3 | 0.21 | 0.43 | 0 | 28 | 47.30326 |
| 0.07 | 0.5 | 2.99 | 0.31 | 0.05 | 1.51 | 7 | 22.50834 |
| 0.14 | 0.5 | 2.99 | 0.3 | 0.11 | 1.51 | 7 | 24.0295 |
| 0.22 | 0.5 | 2.99 | 0.28 | 0.18 | 1.51 | 7 | 25.59032 |
| 0.29 | 0.5 | 2.99 | 0.26 | 0.25 | 1.51 | 7 | 25.33658 |
| 0 | 0.42 | 2.53 | 0.33 | 0 | 3.67 | 7 | 27.23661 |
| 0.06 | 0.42 | 2.53 | 0.31 | 0.05 | 3.67 | 7 | 29.00095 |
| 0 | 0.42 | 2.53 | 0.33 | 0 | 3.67 | 28 | 39.23225 |
| 0.06 | 0.42 | 2.53 | 0.31 | 0.05 | 3.67 | 28 | 42.41561 |
| 0.12 | 0.42 | 2.53 | 0.3 | 0.11 | 3.67 | 28 | 44.63073 |
| 0.25 | 0.42 | 2.53 | 0.26 | 0.25 | 3.67 | 28 | 44.52344 |
| 0.07 | 0.5 | 2.99 | 0.31 | 0.05 | 1.51 | 90 | 38.58288 |
| 0.22 | 0.5 | 2.99 | 0.28 | 0.18 | 1.51 | 90 | 41.82162 |
| 0.29 | 0.5 | 2.99 | 0.26 | 0.25 | 1.51 | 90 | 41.09792 |
| 0 | 0.42 | 2.53 | 0.33 | 0 | 3.67 | 90 | 43.30153 |
| 0.18 | 0.42 | 2.53 | 0.28 | 0.18 | 3.67 | 90 | 47.71931 |
| 0.12 | 0.42 | 2.53 | 0.3 | 0.11 | 3.67 | 365 | 52.48056 |
| 0.25 | 0.42 | 2.53 | 0.26 | 0.25 | 3.67 | 365 | 53.01538 |
| 0.19 | 0.45 | 2.43 | 0.4 | 0.11 | 0 | 7 | 32.15514 |
| 0 | 0.45 | 2.43 | 0.44 | 0 | 0 | 28 | 45.81025 |
| 0.58 | 0.45 | 2.43 | 0.31 | 0.43 | 0 | 28 | 45.1878 |

Table S2: Dataset used in the current study for elastic modulus

| WFS/C | W/C | CA/C | FA/TA | WFS/FA | 1000SP/C | Age | E (GPa) |
| --- | --- | --- | --- | --- | --- | --- | --- |
| 0 | 0.5 | 3.3 | 0.3 | 0 | 12.1 | 7 | 22 |
| 0.29 | 0.5 | 3.3 | 0.24 | 0.25 | 15.86 | 7 | 23.8 |
| 0.15 | 0.5 | 3.3 | 0.27 | 0.11 | 15.86 | 28 | 26.75 |
| 0.29 | 0.5 | 3.3 | 0.24 | 0.25 | 15.86 | 28 | 27.6 |
| 0 | 0.5 | 3.3 | 0.3 | 0 | 12.1 | 56 | 26.4 |
| 0.15 | 0.5 | 3.3 | 0.27 | 0.11 | 15.86 | 56 | 28.4 |
| 0 | 0.5 | 3.3 | 0.3 | 0 | 12.1 | 90 | 27.1 |
| 0.15 | 0.5 | 3.3 | 0.27 | 0.11 | 15.86 | 90 | 29.2 |
| 0.29 | 0.5 | 3.3 | 0.24 | 0.25 | 15.86 | 90 | 30 |
| 0.29 | 0.5 | 3.3 | 0.24 | 0.25 | 15.86 | 365 | 30.6 |
| 0.44 | 0.5 | 3.3 | 0.21 | 0.43 | 15.86 | 365 | 31.8 |
| 0 | 0.4 | 2.53 | 0.33 | 0 | 3.67 | 28 | 29.9 |
| 0.12 | 0.4 | 2.53 | 0.3 | 0.11 | 3.67 | 28 | 31.4 |
| 0.18 | 0.4 | 2.53 | 0.28 | 0.18 | 3.67 | 28 | 31.8 |
| 0 | 0.4 | 2.53 | 0.33 | 0 | 3.67 | 90 | 31.7 |
| 0.06 | 0.4 | 2.53 | 0.31 | 0.05 | 3.67 | 90 | 32.5 |
| 0.12 | 0.4 | 2.53 | 0.3 | 0.11 | 3.67 | 90 | 32.9 |
| 0.18 | 0.4 | 2.53 | 0.28 | 0.18 | 3.67 | 90 | 33.6 |
| 0.25 | 0.4 | 2.53 | 0.26 | 0.25 | 3.67 | 90 | 33.3 |
| 0 | 0.5 | 3.13 | 0.33 | 0 | 0 | 7 | 20.41 |
| 0.08 | 0.5 | 3.13 | 0.31 | 0.08 | 0 | 7 | 20.81 |
| 0.15 | 0.5 | 3.13 | 0.3 | 0.15 | 0 | 7 | 21.22 |
| 0.38 | 0.5 | 3.13 | 0.25 | 0.38 | 0 | 7 | 20.4 |
| 0 | 0.5 | 3.13 | 0.33 | 0 | 0 | 28 | 23.6 |
| 0.08 | 0.5 | 3.13 | 0.31 | 0.08 | 0 | 28 | 24.24 |
| 0.23 | 0.5 | 3.13 | 0.28 | 0.23 | 0 | 28 | 25.19 |
| 0.31 | 0.5 | 3.13 | 0.26 | 0.31 | 0 | 28 | 25.4 |
| 0 | 0.5 | 3.13 | 0.33 | 0 | 0 | 90 | 24.83 |
| 0.08 | 0.5 | 3.13 | 0.31 | 0.08 | 0 | 90 | 25.01 |
| 0.15 | 0.5 | 3.13 | 0.3 | 0.15 | 0 | 90 | 25.19 |
| 0.38 | 0.5 | 3.13 | 0.25 | 0.38 | 0 | 90 | 24.6 |
| 0 | 0.4 | 2.13 | 0.5 | 0 | 0 | 7 | 35.12 |
| 0.21 | 0.4 | 1.92 | 0.5 | 0.1 | 0 | 7 | 37 |
| 0.64 | 0.4 | 1.49 | 0.5 | 0.3 | 0 | 7 | 38.13 |
| 0 | 0.4 | 2.13 | 0.5 | 0 | 0 | 7 | 35.12 |
| 0.21 | 0.4 | 2.13 | 0.45 | 0.11 | 0 | 7 | 37.98 |
| 0.43 | 0.4 | 2.13 | 0.4 | 0.25 | 0 | 7 | 39.9 |
| 0 | 0.4 | 2.13 | 0.5 | 0 | 0 | 7 | 35.04 |
| 0.43 | 0.4 | 1.92 | 0.45 | 0.22 | 0 | 7 | 34.89 |
| 1.28 | 0.4 | 1.49 | 0.35 | 0.86 | 0 | 7 | 22.81 |
| 0.21 | 0.4 | 1.92 | 0.5 | 0.1 | 0 | 14 | 41.03 |
| 0.43 | 0.4 | 1.71 | 0.5 | 0.2 | 0 | 14 | 41.87 |
| 0.64 | 0.4 | 1.49 | 0.5 | 0.3 | 0 | 14 | 42.32 |
| 0 | 0.4 | 2.13 | 0.5 | 0 | 0 | 14 | 40.39 |
| 0.21 | 0.4 | 2.13 | 0.45 | 0.11 | 0 | 14 | 42.02 |
| 0.64 | 0.4 | 2.13 | 0.35 | 0.43 | 0 | 14 | 44.48 |
| 0 | 0.4 | 2.13 | 0.5 | 0 | 0 | 14 | 40.44 |
| 0.43 | 0.4 | 1.92 | 0.45 | 0.22 | 0 | 14 | 40.07 |
| 0.85 | 0.4 | 1.71 | 0.4 | 0.5 | 0 | 14 | 30.74 |
| 0 | 0.4 | 2.13 | 0.5 | 0 | 0 | 28 | 42.81 |
| 0.21 | 0.4 | 1.92 | 0.5 | 0.1 | 0 | 28 | 43.1 |
| 0.64 | 0.4 | 1.49 | 0.5 | 0.3 | 0 | 28 | 45.12 |
| 0 | 0.4 | 2.13 | 0.5 | 0 | 0 | 28 | 42.81 |
| 0.43 | 0.4 | 2.13 | 0.4 | 0.25 | 0 | 28 | 45.67 |
| 0.64 | 0.4 | 2.13 | 0.35 | 0.43 | 0 | 28 | 46.65 |
| 0 | 0.4 | 2.13 | 0.5 | 0 | 0 | 28 | 42.81 |
| 0.43 | 0.4 | 1.92 | 0.45 | 0.22 | 0 | 28 | 43.11 |
| 0 | 0.48 | 2.97 | 0.44 | 0 | 0 | 3 | 22.5 |
| 0.83 | 0.48 | 2.97 | 0.34 | 0.54 | 0 | 3 | 18.4 |
| 0.59 | 0.48 | 2.97 | 0.37 | 0.33 | 0 | 3 | 23.9 |
| 0 | 0.48 | 2.97 | 0.44 | 0 | 0 | 7 | 24.3 |
| 0.59 | 0.48 | 2.97 | 0.37 | 0.33 | 0 | 7 | 24.5 |
| 0.59 | 0.48 | 2.97 | 0.37 | 0.33 | 0 | 7 | 25 |
| 0.83 | 0.48 | 2.97 | 0.34 | 0.54 | 0 | 7 | 27.6 |
| 0 | 0.48 | 2.97 | 0.44 | 0 | 0 | 28 | 31.7 |
| 0.83 | 0.48 | 2.97 | 0.34 | 0.54 | 0 | 28 | 32.6 |
| 0.59 | 0.48 | 2.97 | 0.37 | 0.33 | 0 | 28 | 33.4 |
| 0.83 | 0.48 | 2.97 | 0.34 | 0.54 | 0 | 28 | 33.3 |
| 0.15 | 0.55 | 3 | 0.3 | 0.11 | 0 | 28 | 30 |
| 1.05 | 0.55 | 3 | 0.1 | 2.33 | 0 | 28 | 30.5 |
| 0 | 0.45 | 3 | 0.25 | 0 | 0 | 28 | 34.5 |
| 0.15 | 0.45 | 3 | 0.24 | 0.11 | 0 | 28 | 37 |
| 0.45 | 0.45 | 3 | 0.21 | 0.43 | 0 | 28 | 35 |
| 0.75 | 0.45 | 3 | 0.17 | 1 | 0 | 28 | 35.5 |
| 1.05 | 0.45 | 3 | 0.12 | 2.33 | 0 | 28 | 34.5 |
| 0 | 0.5 | 2.99 | 0.33 | 0 | 1.51 | 7 | 20.5 |
| 0.14 | 0.5 | 2.99 | 0.3 | 0.11 | 1.51 | 7 | 21.3 |
| 0.22 | 0.5 | 2.99 | 0.28 | 0.18 | 1.51 | 7 | 21.9 |
| 0.29 | 0.5 | 2.99 | 0.26 | 0.25 | 1.51 | 7 | 21.5 |
| 0 | 0.42 | 2.53 | 0.33 | 0 | 3.67 | 7 | 25.7 |
| 0.06 | 0.42 | 2.53 | 0.31 | 0.05 | 3.67 | 7 | 26.6 |
| 0.12 | 0.42 | 2.53 | 0.3 | 0.11 | 3.67 | 7 | 27.1 |
| 0.18 | 0.42 | 2.53 | 0.28 | 0.18 | 3.67 | 7 | 27.7 |
| 0.25 | 0.42 | 2.53 | 0.26 | 0.25 | 3.67 | 7 | 27.2 |
| 0.22 | 0.5 | 2.99 | 0.28 | 0.18 | 1.51 | 28 | 25.2 |
| 0 | 0.42 | 2.53 | 0.33 | 0 | 3.67 | 28 | 29.9 |
| 0.06 | 0.42 | 2.53 | 0.31 | 0.05 | 3.67 | 28 | 30.4 |
| 0.12 | 0.42 | 2.53 | 0.3 | 0.11 | 3.67 | 28 | 30.77 |
| 0.18 | 0.42 | 2.53 | 0.28 | 0.18 | 3.67 | 28 | 31.34 |
| 0.07 | 0.5 | 2.99 | 0.31 | 0.05 | 1.51 | 90 | 24.9 |
| 0.22 | 0.5 | 2.99 | 0.28 | 0.18 | 1.51 | 90 | 25.8 |
| 0.29 | 0.5 | 2.99 | 0.26 | 0.25 | 1.51 | 90 | 25.7 |
| 0 | 0.42 | 2.53 | 0.33 | 0 | 3.67 | 90 | 30.6 |
| 0.06 | 0.42 | 2.53 | 0.31 | 0.05 | 3.67 | 90 | 31 |
| 0.12 | 0.42 | 2.53 | 0.3 | 0.11 | 3.67 | 90 | 31.79 |
| 0.18 | 0.42 | 2.53 | 0.28 | 0.18 | 3.67 | 90 | 32.19 |
| 0 | 0.5 | 2.99 | 0.33 | 0 | 1.51 | 365 | 25.8 |
| 0.07 | 0.5 | 2.99 | 0.31 | 0.05 | 1.51 | 365 | 26.4 |
| 0.14 | 0.5 | 2.99 | 0.3 | 0.11 | 1.51 | 365 | 27.2 |
| 0.29 | 0.5 | 2.99 | 0.26 | 0.25 | 1.51 | 365 | 25.7 |
| 0 | 0.42 | 2.53 | 0.33 | 0 | 3.67 | 365 | 32.3 |
| 0.18 | 0.42 | 2.53 | 0.28 | 0.18 | 3.67 | 365 | 34.1 |
| 0.15 | 0.5 | 3.3 | 0.27 | 0.11 | 15.86 | 7 | 23.6 |
| 0.44 | 0.5 | 3.3 | 0.21 | 0.43 | 15.86 | 7 | 24.2 |
| 0 | 0.5 | 3.3 | 0.3 | 0 | 12.1 | 28 | 25.1 |
| 0.44 | 0.5 | 3.3 | 0.21 | 0.43 | 15.86 | 28 | 28.4 |
| 0.29 | 0.5 | 3.3 | 0.24 | 0.25 | 15.86 | 56 | 29.3 |
| 0.44 | 0.5 | 3.3 | 0.21 | 0.43 | 15.86 | 56 | 30.3 |
| 0.44 | 0.5 | 3.3 | 0.21 | 0.43 | 15.86 | 90 | 31.2 |
| 0 | 0.5 | 3.3 | 0.3 | 0 | 12.1 | 365 | 27.7 |
| 0.15 | 0.5 | 3.3 | 0.27 | 0.11 | 15.86 | 365 | 29.5 |
| 0.06 | 0.4 | 2.53 | 0.31 | 0.05 | 3.67 | 28 | 30.4 |
| 0.25 | 0.4 | 2.53 | 0.26 | 0.25 | 3.67 | 28 | 31.2 |
| 0.23 | 0.5 | 3.13 | 0.28 | 0.23 | 0 | 7 | 21.8 |
| 0.31 | 0.5 | 3.13 | 0.26 | 0.31 | 0 | 7 | 22.21 |
| 0.15 | 0.5 | 3.13 | 0.3 | 0.15 | 0 | 28 | 24.78 |
| 0.38 | 0.5 | 3.13 | 0.25 | 0.38 | 0 | 28 | 23.43 |
| 0.23 | 0.5 | 3.13 | 0.28 | 0.23 | 0 | 90 | 25.59 |
| 0.31 | 0.5 | 3.13 | 0.26 | 0.31 | 0 | 90 | 25.73 |
| 0.43 | 0.4 | 1.71 | 0.5 | 0.2 | 0 | 7 | 37.44 |
| 0.64 | 0.4 | 2.13 | 0.35 | 0.43 | 0 | 7 | 41.08 |
| 0.85 | 0.4 | 1.71 | 0.4 | 0.5 | 0 | 7 | 27.19 |
| 0 | 0.4 | 2.13 | 0.5 | 0 | 0 | 14 | 40.44 |
| 0.43 | 0.4 | 2.13 | 0.4 | 0.25 | 0 | 14 | 43.05 |
| 1.28 | 0.4 | 1.49 | 0.35 | 0.86 | 0 | 14 | 26.3 |
| 0.43 | 0.4 | 1.71 | 0.5 | 0.2 | 0 | 28 | 44.09 |
| 0.21 | 0.4 | 2.13 | 0.45 | 0.11 | 0 | 28 | 44.33 |
| 0.85 | 0.4 | 1.71 | 0.4 | 0.5 | 0 | 28 | 36.22 |
| 1.28 | 0.4 | 1.49 | 0.35 | 0.86 | 0 | 28 | 32.15 |
| 0.59 | 0.48 | 2.97 | 0.37 | 0.33 | 0 | 3 | 20.2 |
| 0.83 | 0.48 | 2.97 | 0.34 | 0.54 | 0 | 3 | 20.9 |
| 0.83 | 0.48 | 2.97 | 0.34 | 0.54 | 0 | 7 | 23.4 |
| 0.59 | 0.48 | 2.97 | 0.37 | 0.33 | 0 | 28 | 31.7 |
| 0 | 0.55 | 3 | 0.33 | 0 | 0 | 28 | 30 |
| 0.45 | 0.55 | 3 | 0.23 | 0.43 | 0 | 28 | 30.5 |
| 0.75 | 0.55 | 3 | 0.17 | 1 | 0 | 28 | 29.5 |
| 0.07 | 0.5 | 2.99 | 0.31 | 0.05 | 1.51 | 7 | 21.1 |
| 0 | 0.5 | 2.99 | 0.33 | 0 | 1.51 | 28 | 23.8 |
| 0.25 | 0.42 | 2.53 | 0.26 | 0.25 | 3.67 | 28 | 31.07 |
| 0 | 0.5 | 2.99 | 0.33 | 0 | 1.51 | 90 | 24.6 |
| 0.14 | 0.5 | 2.99 | 0.3 | 0.11 | 1.51 | 90 | 25.4 |
| 0.25 | 0.42 | 2.53 | 0.26 | 0.25 | 3.67 | 90 | 31.98 |
| 0.22 | 0.5 | 2.99 | 0.28 | 0.18 | 1.51 | 365 | 27.5 |
| 0.06 | 0.42 | 2.53 | 0.31 | 0.05 | 3.67 | 365 | 32.9 |
| 0.12 | 0.42 | 2.53 | 0.3 | 0.11 | 3.67 | 365 | 33.71 |
| 0.25 | 0.42 | 2.53 | 0.26 | 0.25 | 3.67 | 365 | 33.6 |

Table S3: Dataset used in the current study for split tensile strength

| WFS/C | W/C | CA/C | FA/TA | WFS/FA | 1000SP/C | Age | STS (MPa) |
| --- | --- | --- | --- | --- | --- | --- | --- |
| 0.17 | 0.5 | 3.6 | 0.29 | 0.11 | 5 | 28 | 1.84 |
| 0.35 | 0.5 | 3.6 | 0.26 | 0.25 | 5 | 28 | 1.98 |
| 0.52 | 0.5 | 3.6 | 0.23 | 0.43 | 5 | 28 | 2.58 |
| 0.86 | 0.53 | 3.6 | 0.16 | 1 | 5 | 28 | 2.38 |
| 1.04 | 0.56 | 3.6 | 0.13 | 1.5 | 5 | 28 | 1.72 |
| 0 | 0.5 | 3.6 | 0.32 | 0 | 5 | 90 | 2.66 |
| 0.17 | 0.5 | 3.6 | 0.29 | 0.11 | 5 | 90 | 2.35 |
| 0.52 | 0.5 | 3.6 | 0.23 | 0.43 | 5 | 90 | 3.33 |
| 0.69 | 0.51 | 3.6 | 0.19 | 0.67 | 5 | 90 | 3.26 |
| 0.86 | 0.53 | 3.6 | 0.16 | 1 | 5 | 90 | 3.19 |
| 1.04 | 0.56 | 3.6 | 0.13 | 1.5 | 5 | 90 | 2.16 |
| 0.17 | 0.5 | 3.6 | 0.29 | 0.11 | 5 | 365 | 2.66 |
| 0.35 | 0.5 | 3.6 | 0.26 | 0.25 | 5 | 365 | 2.86 |
| 0.52 | 0.5 | 3.6 | 0.23 | 0.43 | 5 | 365 | 3.5 |
| 0.69 | 0.51 | 3.6 | 0.19 | 0.67 | 5 | 365 | 3.42 |
| 1.04 | 0.56 | 3.6 | 0.13 | 1.5 | 5 | 365 | 2.52 |
| 0.13 | 0.51 | 2.88 | 0.22 | 0.11 | 17.8 | 7 | 4.1 |
| 0.38 | 0.53 | 2.88 | 0.17 | 0.43 | 17.8 | 7 | 3.4 |
| 0 | 0.46 | 2.88 | 0.24 | 0 | 17.8 | 28 | 4.6 |
| 0.13 | 0.51 | 2.88 | 0.22 | 0.11 | 17.8 | 28 | 4.2 |
| 0.51 | 0.54 | 2.88 | 0.1 | 1 | 17.8 | 28 | 3.7 |
| 0.13 | 0.51 | 2.88 | 0.22 | 0.11 | 17.8 | 56 | 4.4 |
| 0.25 | 0.52 | 2.88 | 0.2 | 0.25 | 17.8 | 56 | 4.1 |
| 0.38 | 0.53 | 2.88 | 0.17 | 0.43 | 17.8 | 56 | 3.9 |
| 0.51 | 0.54 | 2.88 | 0.1 | 1 | 17.8 | 56 | 3.83 |
| 0 | 0.46 | 2.88 | 0.24 | 0 | 17.8 | 90 | 4.85 |
| 0.13 | 0.51 | 2.88 | 0.22 | 0.11 | 17.8 | 90 | 4.5 |
| 0.25 | 0.52 | 2.88 | 0.2 | 0.25 | 17.8 | 90 | 4.15 |
| 0 | 0.5 | 3.3 | 0.3 | 0 | 12.1 | 7 | 1.89 |
| 0.15 | 0.5 | 3.3 | 0.27 | 0.11 | 15.86 | 7 | 1.96 |
| 0.29 | 0.5 | 3.3 | 0.24 | 0.25 | 15.86 | 7 | 2.05 |
| 0.44 | 0.5 | 3.3 | 0.21 | 0.43 | 15.86 | 7 | 2.08 |
| 0.15 | 0.5 | 3.3 | 0.27 | 0.11 | 15.86 | 28 | 2.85 |
| 0.29 | 0.5 | 3.3 | 0.24 | 0.25 | 15.86 | 28 | 2.9 |
| 0.44 | 0.5 | 3.3 | 0.21 | 0.43 | 15.86 | 28 | 3 |
| 0 | 0.5 | 3.3 | 0.3 | 0 | 12.1 | 56 | 2.93 |
| 0.44 | 0.5 | 3.3 | 0.21 | 0.43 | 15.86 | 56 | 3.24 |
| 0 | 0.5 | 3.3 | 0.3 | 0 | 12.1 | 90 | 2.99 |
| 0.15 | 0.5 | 3.3 | 0.27 | 0.11 | 15.86 | 90 | 3.13 |
| 0.29 | 0.5 | 3.3 | 0.24 | 0.25 | 15.86 | 90 | 3.21 |
| 0 | 0.5 | 3.3 | 0.3 | 0 | 12.1 | 365 | 3.1 |
| 0.29 | 0.5 | 3.3 | 0.24 | 0.25 | 15.86 | 365 | 3.32 |
| 0 | 0.4 | 2.53 | 0.33 | 0 | 3.67 | 7 | 2.77 |
| 0.06 | 0.4 | 2.53 | 0.31 | 0.05 | 3.67 | 7 | 3.09 |
| 0 | 0.4 | 2.53 | 0.33 | 0 | 3.67 | 28 | 4.23 |
| 0.25 | 0.4 | 2.53 | 0.26 | 0.25 | 3.67 | 28 | 4.51 |
| 0 | 0.4 | 2.53 | 0.33 | 0 | 3.67 | 90 | 4.32 |
| 0.06 | 0.4 | 2.53 | 0.31 | 0.05 | 3.67 | 90 | 4.45 |
| 0 | 0.5 | 3.13 | 0.33 | 0 | 0 | 7 | 1.8 |
| 0.23 | 0.5 | 3.13 | 0.28 | 0.23 | 0 | 7 | 2.1 |
| 0.31 | 0.5 | 3.13 | 0.26 | 0.31 | 0 | 7 | 2.2 |
| 0.38 | 0.5 | 3.13 | 0.25 | 0.38 | 0 | 7 | 1.8 |
| 0.31 | 0.5 | 3.13 | 0.26 | 0.31 | 0 | 28 | 2.8 |
| 0.38 | 0.5 | 3.13 | 0.25 | 0.38 | 0 | 28 | 2.1 |
| 0 | 0.44 | 2.86 | 0.35 | 0 | 0 | 7 | 2.77 |
| 0.31 | 0.44 | 2.86 | 0.3 | 0.25 | 0 | 7 | 2.6 |
| 0.61 | 0.44 | 2.86 | 0.24 | 0.67 | 0 | 7 | 2.31 |
| 0.77 | 0.44 | 2.86 | 0.21 | 1 | 0 | 7 | 2.21 |
| 0 | 0.44 | 2.86 | 0.35 | 0 | 0 | 28 | 2.81 |
| 0.15 | 0.44 | 2.86 | 0.33 | 0.11 | 0 | 28 | 2.76 |
| 0.31 | 0.44 | 2.86 | 0.3 | 0.25 | 0 | 28 | 2.67 |
| 0.46 | 0.44 | 2.86 | 0.27 | 0.43 | 0 | 28 | 2.6 |
| 0.77 | 0.44 | 2.86 | 0.21 | 1 | 0 | 28 | 2.32 |
| 0 | 0.44 | 2.86 | 0.35 | 0 | 0 | 90 | 2.81 |
| 0.31 | 0.44 | 2.86 | 0.3 | 0.25 | 0 | 90 | 2.68 |
| 0.77 | 0.44 | 2.86 | 0.21 | 1 | 0 | 90 | 2.33 |
| 0 | 0.44 | 2.86 | 0.35 | 0 | 0 | 180 | 2.73 |
| 0.15 | 0.44 | 2.86 | 0.33 | 0.11 | 0 | 180 | 2.66 |
| 0.31 | 0.44 | 2.86 | 0.3 | 0.25 | 0 | 180 | 2.66 |
| 0.46 | 0.44 | 2.86 | 0.27 | 0.43 | 0 | 180 | 2.59 |
| 0.61 | 0.44 | 2.86 | 0.24 | 0.67 | 0 | 180 | 2.46 |
| 0.77 | 0.44 | 2.86 | 0.21 | 1 | 0 | 180 | 2.44 |
| 0 | 0.47 | 2.47 | 0.38 | 0 | 0 | 28 | 2.75 |
| 0.31 | 0.47 | 2.47 | 0.33 | 0.25 | 0 | 28 | 2.98 |
| 0.61 | 0.47 | 2.47 | 0.27 | 0.67 | 0 | 28 | 3.4 |
| 0.77 | 0.47 | 2.47 | 0.24 | 1 | 0 | 28 | 3.12 |
| 0.68 | 0.47 | 2.75 | 0.26 | 0.67 | 0 | 28 | 3.04 |
| 0.77 | 0.47 | 3.09 | 0.26 | 0.67 | 0 | 28 | 2.91 |
| 0.65 | 0.47 | 2.6 | 0.27 | 0.67 | 0 | 28 | 3.61 |
| 0.66 | 0.47 | 2.67 | 0.27 | 0.67 | 0 | 28 | 3.67 |
| 0 | 0.52 | 4.2 | 0.38 | 0 | 6 | 28 | 3.3 |
| 0.25 | 0.52 | 4.2 | 0.34 | 0.11 | 6 | 28 | 1.87 |
| 0.51 | 0.52 | 4.2 | 0.3 | 0.25 | 6 | 28 | 2.85 |
| 0.03 | 0.52 | 4.2 | 0.35 | 0.01 | 6 | 28 | 2.08 |
| 0.06 | 0.52 | 4.2 | 0.32 | 0.03 | 6 | 28 | 2.64 |
| 0 | 0.4 | 2.13 | 0.5 | 0 | 0 | 7 | 3.13 |
| 0.21 | 0.4 | 1.92 | 0.5 | 0.1 | 0 | 7 | 3.15 |
| 0.43 | 0.4 | 1.71 | 0.5 | 0.2 | 0 | 7 | 3.23 |
| 0.21 | 0.4 | 2.13 | 0.45 | 0.11 | 0 | 7 | 3.26 |
| 0.43 | 0.4 | 2.13 | 0.4 | 0.25 | 0 | 7 | 3.44 |
| 0.64 | 0.4 | 2.13 | 0.35 | 0.43 | 0 | 7 | 3.63 |
| 0 | 0.4 | 2.13 | 0.5 | 0 | 0 | 7 | 3.13 |
| 0.85 | 0.4 | 1.71 | 0.4 | 0.5 | 0 | 7 | 2.18 |
| 1.28 | 0.4 | 1.49 | 0.35 | 0.86 | 0 | 7 | 1.69 |
| 0 | 0.4 | 2.13 | 0.5 | 0 | 0 | 14 | 3.59 |
| 0.21 | 0.4 | 1.92 | 0.5 | 0.1 | 0 | 14 | 3.69 |
| 0.64 | 0.4 | 1.49 | 0.5 | 0.3 | 0 | 14 | 3.92 |
| 0.21 | 0.4 | 2.13 | 0.45 | 0.11 | 0 | 14 | 3.78 |
| 0.64 | 0.4 | 2.13 | 0.35 | 0.43 | 0 | 14 | 4.23 |
| 0 | 0.4 | 2.13 | 0.5 | 0 | 0 | 14 | 3.59 |
| 0.85 | 0.4 | 1.71 | 0.4 | 0.5 | 0 | 14 | 2.66 |
| 1.28 | 0.4 | 1.49 | 0.35 | 0.86 | 0 | 14 | 2.25 |
| 0 | 0.4 | 2.13 | 0.5 | 0 | 0 | 28 | 4.03 |
| 0.21 | 0.4 | 1.92 | 0.5 | 0.1 | 0 | 28 | 4.15 |
| 0 | 0.4 | 2.13 | 0.5 | 0 | 0 | 28 | 4.03 |
| 0.21 | 0.4 | 2.13 | 0.45 | 0.11 | 0 | 28 | 4.26 |
| 0.43 | 0.4 | 2.13 | 0.4 | 0.25 | 0 | 28 | 4.43 |
| 0.43 | 0.4 | 1.92 | 0.45 | 0.22 | 0 | 28 | 3.95 |
| 0.85 | 0.4 | 1.71 | 0.4 | 0.5 | 0 | 28 | 3.26 |
| 1.28 | 0.4 | 1.49 | 0.35 | 0.86 | 0 | 28 | 2.93 |
| 0 | 0.48 | 2.97 | 0.44 | 0 | 0 | 3 | 2.7 |
| 0.59 | 0.48 | 2.97 | 0.37 | 0.33 | 0 | 3 | 1.8 |
| 0.83 | 0.48 | 2.97 | 0.34 | 0.54 | 0 | 3 | 2.1 |
| 0.59 | 0.48 | 2.97 | 0.37 | 0.33 | 0 | 3 | 2.3 |
| 0.83 | 0.48 | 2.97 | 0.34 | 0.54 | 0 | 3 | 2.1 |
| 0.59 | 0.48 | 2.97 | 0.37 | 0.33 | 0 | 7 | 3.2 |
| 0.59 | 0.48 | 2.97 | 0.37 | 0.33 | 0 | 7 | 3.5 |
| 0.83 | 0.48 | 2.97 | 0.34 | 0.54 | 0 | 7 | 2.5 |
| 0.59 | 0.48 | 2.97 | 0.37 | 0.33 | 0 | 28 | 3.6 |
| 0.59 | 0.48 | 2.97 | 0.37 | 0.33 | 0 | 28 | 4 |
| 0 | 0.4 | 2.5 | 0.29 | 0 | 0 | 28 | 4.3 |
| 0.6 | 0.4 | 2.5 | 0.14 | 1.46 | 0 | 28 | 3.15 |
| 0 | 0.55 | 2.16 | 0.44 | 0 | 0 | 7 | 3.13 |
| 0.17 | 0.55 | 2.16 | 0.42 | 0.11 | 0 | 7 | 2.93 |
| 0.35 | 0.55 | 2.16 | 0.4 | 0.24 | 0 | 7 | 3 |
| 0.71 | 0.55 | 2.16 | 0.33 | 0.67 | 0 | 7 | 3.21 |
| 0.9 | 0.55 | 2.16 | 0.29 | 1 | 0 | 7 | 3.33 |
| 0 | 0.55 | 2.16 | 0.44 | 0 | 0 | 28 | 3.51 |
| 0.17 | 0.55 | 2.16 | 0.42 | 0.11 | 0 | 28 | 3.73 |
| 0.35 | 0.55 | 2.16 | 0.4 | 0.24 | 0 | 28 | 3.59 |
| 0.71 | 0.55 | 2.16 | 0.33 | 0.67 | 0 | 28 | 3.99 |
| 0.18 | 0.5 | 3.38 | 0.33 | 0.11 | 0 | 7 | 1.92 |
| 0.36 | 0.5 | 3.38 | 0.3 | 0.25 | 0 | 7 | 2.01 |
| 0.72 | 0.5 | 3.38 | 0.24 | 0.67 | 0 | 7 | 2.02 |
| 0.18 | 0.5 | 3.38 | 0.33 | 0.11 | 0 | 28 | 2.45 |
| 0.36 | 0.5 | 3.38 | 0.3 | 0.25 | 0 | 28 | 2.54 |
| 0.72 | 0.5 | 3.38 | 0.24 | 0.67 | 0 | 28 | 2.52 |
| 0 | 0.5 | 3.38 | 0.35 | 0 | 0 | 56 | 2.92 |
| 0.18 | 0.5 | 3.38 | 0.33 | 0.11 | 0 | 56 | 3.04 |
| 0.36 | 0.5 | 3.38 | 0.3 | 0.25 | 0 | 56 | 3.18 |
| 0.54 | 0.5 | 3.38 | 0.27 | 0.43 | 0 | 56 | 3.31 |
| 0.72 | 0.5 | 3.38 | 0.24 | 0.67 | 0 | 56 | 3.09 |
| 0 | 0.5 | 3.38 | 0.35 | 0 | 0 | 90 | 3.59 |
| 0.54 | 0.5 | 3.38 | 0.27 | 0.43 | 0 | 90 | 4.07 |
| 0.72 | 0.5 | 3.38 | 0.24 | 0.67 | 0 | 90 | 3.87 |
| 0 | 0.43 | 2.57 | 0.41 | 0 | 0 | 7 | 2.2 |
| 0.18 | 0.43 | 2.99 | 0.35 | 0.11 | 0 | 7 | 2.58 |
| 0.27 | 0.43 | 2.99 | 0.34 | 0.18 | 0 | 7 | 2.53 |
| 0.36 | 0.43 | 2.99 | 0.32 | 0.25 | 0 | 7 | 2.16 |
| 0.15 | 0.43 | 2.8 | 0.32 | 0.11 | 0 | 7 | 2.72 |
| 0.29 | 0.43 | 2.8 | 0.3 | 0.25 | 0 | 7 | 2.12 |
| 0 | 0.43 | 2.57 | 0.41 | 0 | 0 | 28 | 3.3 |
| 0.18 | 0.43 | 2.99 | 0.35 | 0.11 | 0 | 28 | 3.87 |
| 0.27 | 0.43 | 2.99 | 0.34 | 0.18 | 0 | 28 | 3.77 |
| 0.36 | 0.43 | 2.99 | 0.32 | 0.25 | 0 | 28 | 2.93 |
| 0 | 0.43 | 2.8 | 0.34 | 0 | 0 | 28 | 3.21 |
| 0.29 | 0.43 | 2.8 | 0.3 | 0.25 | 0 | 28 | 3.25 |
| 0.39 | 0.42 | 3.16 | 0.33 | 0.25 | 0 | 7 | 2.2 |
| 0.78 | 0.42 | 3.16 | 0.27 | 0.67 | 0 | 7 | 3.26 |
| 1.17 | 0.42 | 3.16 | 0.2 | 1.5 | 0 | 7 | 2.62 |
| 0.95 | 0.42 | 3.33 | 0.38 | 0 | 0 | 7 | 2.83 |
| 0.9 | 0.42 | 3.51 | 0.38 | 0 | 0 | 7 | 2.24 |
| 0.85 | 0.42 | 3.72 | 0.38 | 0 | 0 | 7 | 2.16 |
| 0 | 0.42 | 3.16 | 0.38 | 0 | 0 | 14 | 2.98 |
| 0.39 | 0.42 | 3.16 | 0.33 | 0.25 | 0 | 14 | 3.15 |
| 1.17 | 0.42 | 3.16 | 0.2 | 1.5 | 0 | 14 | 3.36 |
| 0.95 | 0.42 | 3.33 | 0.38 | 0 | 0 | 14 | 3.53 |
| 0.9 | 0.42 | 3.51 | 0.38 | 0 | 0 | 14 | 2.62 |
| 0 | 0.42 | 3.16 | 0.38 | 0 | 0 | 28 | 3.67 |
| 0.39 | 0.42 | 3.16 | 0.33 | 0.25 | 0 | 28 | 3.78 |
| 0.78 | 0.42 | 3.16 | 0.27 | 0.67 | 0 | 28 | 4.16 |
| 0.9 | 0.42 | 3.51 | 0.38 | 0 | 0 | 28 | 3.01 |
| 0.85 | 0.42 | 3.72 | 0.38 | 0 | 0 | 28 | 2.83 |
| 0 | 0.45 | 0.93 | 0.69 | 0 | 0 | 28 | 3.27 |
| 0.42 | 0.45 | 0.93 | 0.69 | 0.25 | 0 | 28 | 3.32 |
| 0.63 | 0.45 | 0.93 | 0.69 | 0.43 | 0 | 28 | 3.14 |
| 0 | 0.45 | 1.9 | 0.51 | 0 | 0 | 28 | 3.31 |
| 0.2 | 0.45 | 1.9 | 0.51 | 0.11 | 0 | 28 | 3.24 |
| 0.4 | 0.45 | 1.9 | 0.51 | 0.25 | 0 | 28 | 3.2 |
| 0.59 | 0.45 | 1.9 | 0.51 | 0.43 | 0 | 28 | 3.15 |
| 0 | 0.53 | 3.18 | 0.33 | 0 | 0 | 28 | 3.08 |
| 0.08 | 0.53 | 3.18 | 0.32 | 0.05 | 0 | 28 | 3.24 |
| 0.24 | 0.53 | 3.18 | 0.28 | 0.18 | 0 | 28 | 3.51 |
| 0.32 | 0.53 | 3.18 | 0.27 | 0.25 | 0 | 28 | 3.63 |
| 0.4 | 0.53 | 3.18 | 0.25 | 0.33 | 0 | 28 | 3.73 |
| 0.48 | 0.53 | 3.18 | 0.23 | 0.43 | 0 | 28 | 3.79 |
| 0.56 | 0.53 | 3.18 | 0.22 | 0.54 | 0 | 28 | 3.91 |
| 0.64 | 0.53 | 3.18 | 0.2 | 0.67 | 0 | 28 | 2.86 |
| 0.17 | 0.52 | 2.93 | 0.33 | 0.11 | 0 | 7 | 2.44 |
| 0.35 | 0.52 | 2.93 | 0.3 | 0.25 | 0 | 7 | 2.82 |
| 0.52 | 0.52 | 2.93 | 0.26 | 0.43 | 0 | 7 | 2.41 |
| 0.69 | 0.52 | 2.93 | 0.22 | 0.67 | 0 | 7 | 2.4 |
| 0 | 0.52 | 2.93 | 0.37 | 0 | 0 | 28 | 3.5 |
| 0.17 | 0.52 | 2.93 | 0.33 | 0.11 | 0 | 28 | 3.85 |
| 0.35 | 0.52 | 2.93 | 0.3 | 0.25 | 0 | 28 | 4.24 |
| 0.52 | 0.52 | 2.93 | 0.26 | 0.43 | 0 | 28 | 3.65 |
| 0.69 | 0.52 | 2.93 | 0.22 | 0.67 | 0 | 28 | 3.6 |
| 0.15 | 0.55 | 3 | 0.3 | 0.11 | 0 | 28 | 2.35 |
| 0.45 | 0.55 | 3 | 0.23 | 0.43 | 0 | 28 | 2.5 |
| 0.75 | 0.55 | 3 | 0.17 | 1 | 0 | 28 | 2.4 |
| 1.05 | 0.55 | 3 | 0.1 | 2.33 | 0 | 28 | 2.6 |
| 0 | 0.45 | 3 | 0.25 | 0 | 0 | 28 | 2.85 |
| 0.45 | 0.45 | 3 | 0.21 | 0.43 | 0 | 28 | 2.9 |
| 0.75 | 0.45 | 3 | 0.17 | 1 | 0 | 28 | 2.9 |
| 1.05 | 0.45 | 3 | 0.12 | 2.33 | 0 | 28 | 2.9 |
| 0 | 0.5 | 2.99 | 0.33 | 0 | 1.51 | 7 | 2.15 |
| 0.14 | 0.5 | 2.99 | 0.3 | 0.11 | 1.51 | 7 | 2.38 |
| 0 | 0.42 | 2.53 | 0.33 | 0 | 3.67 | 7 | 2.77 |
| 0.06 | 0.42 | 2.53 | 0.31 | 0.05 | 3.67 | 7 | 3.1 |
| 0.12 | 0.42 | 2.53 | 0.3 | 0.11 | 3.67 | 7 | 3.2 |
| 0.18 | 0.42 | 2.53 | 0.28 | 0.18 | 3.67 | 7 | 3.28 |
| 0.25 | 0.42 | 2.53 | 0.26 | 0.25 | 3.67 | 7 | 3.1 |
| 0 | 0.5 | 2.99 | 0.33 | 0 | 1.51 | 28 | 4.23 |
| 0.07 | 0.5 | 2.99 | 0.31 | 0.05 | 1.51 | 28 | 4.58 |
| 0.14 | 0.5 | 2.99 | 0.3 | 0.11 | 1.51 | 28 | 4.76 |
| 0.22 | 0.5 | 2.99 | 0.28 | 0.18 | 1.51 | 28 | 4.77 |
| 0 | 0.42 | 2.53 | 0.33 | 0 | 3.67 | 28 | 4.23 |
| 0.06 | 0.42 | 2.53 | 0.31 | 0.05 | 3.67 | 28 | 4.38 |
| 0.12 | 0.42 | 2.53 | 0.3 | 0.11 | 3.67 | 28 | 4.58 |
| 0.18 | 0.42 | 2.53 | 0.28 | 0.18 | 3.67 | 28 | 4.67 |
| 0.25 | 0.42 | 2.53 | 0.26 | 0.25 | 3.67 | 28 | 4.5 |
| 0.07 | 0.5 | 2.99 | 0.31 | 0.05 | 1.51 | 90 | 4.59 |
| 0.29 | 0.5 | 2.99 | 0.26 | 0.25 | 1.51 | 90 | 4.72 |
| 0 | 0.42 | 2.53 | 0.33 | 0 | 3.67 | 90 | 4.31 |
| 0.06 | 0.42 | 2.53 | 0.31 | 0.05 | 3.67 | 90 | 4.45 |
| 0.18 | 0.42 | 2.53 | 0.28 | 0.18 | 3.67 | 90 | 4.8 |
| 0 | 0.5 | 2.99 | 0.33 | 0 | 1.51 | 365 | 3.96 |
| 0.07 | 0.5 | 2.99 | 0.31 | 0.05 | 1.51 | 365 | 4.1 |
| 0.22 | 0.5 | 2.99 | 0.28 | 0.18 | 1.51 | 365 | 4.36 |
| 0.29 | 0.5 | 2.99 | 0.26 | 0.25 | 1.51 | 365 | 4.29 |
| 0 | 0.42 | 2.53 | 0.33 | 0 | 3.67 | 365 | 4.38 |
| 0.06 | 0.42 | 2.53 | 0.31 | 0.05 | 3.67 | 365 | 4.6 |
| 0.12 | 0.42 | 2.53 | 0.3 | 0.11 | 3.67 | 365 | 4.78 |
| 0.25 | 0.42 | 2.53 | 0.26 | 0.25 | 3.67 | 365 | 4.9 |
| 0 | 0.45 | 2.43 | 0.44 | 0 | 0 | 7 | 3.21 |
| 0.19 | 0.45 | 2.43 | 0.4 | 0.11 | 0 | 7 | 3.35 |
| 0.38 | 0.45 | 2.43 | 0.35 | 0.25 | 0 | 7 | 3.48 |
| 0.58 | 0.45 | 2.43 | 0.31 | 0.43 | 0 | 7 | 3.11 |
| 0.77 | 0.45 | 2.43 | 0.26 | 1 | 0 | 7 | 2.83 |
| 0 | 0.45 | 2.43 | 0.44 | 0 | 0 | 28 | 4.53 |
| 0.19 | 0.45 | 2.43 | 0.4 | 0.11 | 0 | 28 | 4.67 |
| 0.58 | 0.45 | 2.43 | 0.31 | 0.43 | 0 | 28 | 4.39 |
